# Falling asleep follows a predictable bifurcation dynamic

**DOI:** 10.1038/s41593-025-02091-1

**Published:** 2025-10-28

**Authors:** Junheng Li, Anastasia Ilina, Robert Peach, Tianyu Wei, Edward Rhodes, Valeria Jaramillo, Ines R. Violante, Mauricio Barahona, Derk-Jan Dijk, Nir Grossman

**Affiliations:** 1 Department of Brain Sciences, Imperial College London, London, UK; 2 UK Dementia Research Institute, Imperial College London, London, UK; 3 Department of Neurology, University Hospital Würzburg, Würzburg, Germany; 4 Department of Mathematics, Imperial College London, London, UK; 5 School of Psychology, Faculty of Health and Medical Sciences, University of Surrey, Guildford, UK; 6 Surrey Sleep Research Centre, University of Surrey, Guildford, UK; 7 UK Dementia Research Institute, Care Research and Technology Centre at Imperial College London, London, UK; 8 University of Surrey, Guilford, UK; 9 School of Biomedical Engineering and Imaging Sciences, King’s College London, London, UK

**Keywords:** Dynamical systems, Sleep

## Abstract

Sleep is a fundamental part of our lives; yet, how our brain falls asleep remains one of the most enduring mysteries of neuroscience. Here we report a new conceptual framework to analyze and model this phenomenon. The framework represents the changes in brain electroencephalogram activity during the transition into sleep as a trajectory in a normalized feature space. We use the framework to show that the brain’s wake-to-sleep transition follows bifurcation dynamics with a distinct tipping point preceded by a critical slowing down. We validate the bifurcation dynamics in two independent datasets, which include more than 1,000 human participants. Finally, we demonstrate the framework’s utility by predicting a person’s progression into sleep in real time with seconds temporal resolution and over 0.95 average accuracy.

## Abstract (web summary)

Li et al. propose a conceptual framework to study the phenomenon of falling asleep based on electroencephalogram data. They show that a tipping point marks the brain’s nonlinear wake-to-sleep transition and that the unfolding process can be tracked in real time.

## Main

Sleep is critical for our physical and mental health. We spend about one-third of our lives asleep^1^. In the nervous system, sleep promotes regenerative processes, including homeostatic plasticity^2^ and functional processes such as memory consolidation^3^. The process of falling asleep is the gateway for these vital physiological and cognitive processes^4^. Falling asleep involves pronounced behavioral and physiological changes such as attenuated response to external stimuli, reduced muscle tone, slowing of respiratory rate and heartrate, and variations in pupil diameter and electrodermal activity^5^. These changes are driven by marked changes in brain activity that can be partially measured noninvasively with scalp electroencephalogram (EEG)^6^. Understanding the dynamics of brain activity during the process of falling asleep has profound implications for human health and public safety, especially given the increasing prevalence of sleep-onset disorders^7^ and the devastating consequences of falling asleep during critical activities such as driving^8^.

Despite the crucial role of sleep in our lives, our understanding of how these marked brain activity changes unfold to yield sleep remains limited^4^. There has been a consensus that falling asleep is a continuous process^5,6^. However, current descriptions of this process are mostly discrete. The classical approach to quantifying changes in brain activity during the transition into sleep is based on Hori, Hayashi and Morikawa’s classification, which assumes a sequential transition between nine distinct EEG patterns, such as alpha wave trains, alpha wave intermittent and vertex sharp waves^9^. More modern analysis approaches quantify the gradual changes in specific EEG features such as the theta and alpha band powers and power ratio, and inter-regional connectivity (via, for example, coherence)^10–18^. Still, they typically classify these feature patterns into discrete microstates (spatiotemporal topographies)^10,18–23^, where the transitional process between wakefulness and bona fide sleep is often referred to as a ‘drowsiness’ state. In practice, these approaches have had limited real-life utility in understanding the phenomenon of falling asleep and supporting the management and treatment of its impairments^5^.

The neurophysiology of wake–sleep transition is governed by mutual inhibitions between wake-promoting and sleep-promoting nuclei, with regulatory inputs from the circadian rhythmicity and sleep homeostasis drives^24^. The mutual inhibition relationship facilitates the fast transition between the states, forming a rapid flip-flop switch in underlying neuronal firing dynamics^24^. The homeostatic and circadian regulation of sleep can be modeled by the classical two-process framework in which sleep propensity results from the interaction of two oscillatory components: process S (sleep homeostasis), dependent on sleep–wake history, and process C (circadian rhythm), which is independent of sleep history^25^. The mutual inhibitions between wake-promoting and sleep-promoting nuclei have been modeled using neural circuit models to describe the dynamics between state transitions, in which sleep propensity is estimated from the average activity of the neural populations^26–28^. Theoretically, these two types of models (that is, the two-process model and neurophysiology model) are related explicitly to each other at the timescale of daily rhythms^29^.

One key insight from these neurophysiological models is that wake–sleep transitions follow a fold (saddle-node) bifurcation dynamic^28,30,31^ (see Supplementary Notes and Supplementary Fig. 1 for an explanation and illustration of a fold bifurcation). Although such bifurcation dynamics have been proposed in theoretical models of sleep–wake transitions, so far there has been no experimental evidence to support such a bifurcation dynamic in the brain (system) between wakefulness and sleep states. We note a case report (two human participants) showing a critical slowing down—a characteristic of the system’s dynamics that can be detected through increased autocorrelation and variance before bifurcation, before sleep stage transitions, albeit not between wake and sleep^32^.

**Fig. 1:**
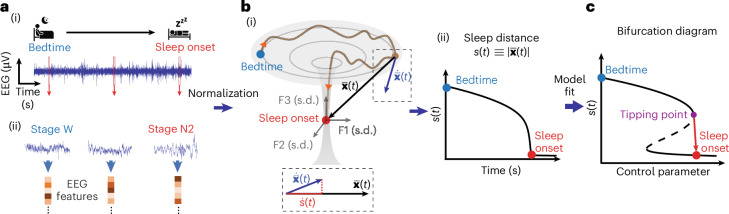
Graphical illustration of the computational framework. **a**, A one-dimensional timeseries EEG signal (i) during falling asleep (transition from bedtime wakefulness to sleep onset) is transformed into a timeseries in the *m*-dimensional feature state space. Here we computed 50 features in 6-s epochs with examples in ii; see Supplementary Table 1 for the feature list. **b**, Feature space trajectory of falling asleep dynamics: (i) an illustration of a simplified three-dimensional feature space showing a bifurcation-like transition (brown curve) between bedtime (blue) and sleep onset (red). The features (F1, F2 and F3) are *z*-score normalized (unit in s.d.), and the space is re-referenced to the sleep onset centroid. The state vector $\,\overline{{\bf{x}}}(t)$ is the instantaneous location; $\dot{\bar{\bf{x}}}(t)$ is the state space velocity $\Delta \bar{\bf{x}}(t)/\Delta t$. Sleep distance is calculated as $s\left(t\right)\equiv \left|\bar{\bf{x}}(t)\right|$, whose dynamics are measured over time (ii). The projection from $\dot{\bar{\bf{x}}}(t)$ to $\bar{\bf{x}}(t)$ is the sleep velocity $\dot{s}\left(t\right)=\dot{\bar{\bf{x}}}\left(t\right)\bullet \hat{x}\left(t\right)$ (dashed box inset, bottom). **c**, By fitting the sleep distance dynamics to a common fold-bifurcation function (model), the bifurcation diagram of the transition can be derived, with a transition tipping point identified (purple).

Here we report a new framework for analyzing and modeling the brain’s falling asleep phenomenon, validate it using two independent datasets and demonstrate its utility by successfully predicting individual sleep progressions in quasi-real time. A graphical illustration of the framework is shown in Fig. 1a–c. The framework is based on transforming the changes in the brain’s EEG activity to a trajectory in a geometrical space of normalized EEG features and monitoring the instantaneous Euclidean distance to the sleep onset location. We show that the timeseries of the so-called sleep distance displays a bifurcation dynamic during the falling asleep process, independent of sleep latency, age or gender, supporting previous theoretical observations. Leveraging bifurcation theory, we then identify a wake–sleep tipping point and demonstrate a critical slowing before the bifurcation, similar to other bifurcation (critical transitions) phenomena in nature^33^. Next, we demonstrate that the coordinates of sleep onset in the feature space are preserved in individual participants across several nights independently of the locations at bedtime in the feature space. We then use the computational framework and preservation of sleep onset coordinates to predict a person’s progression into sleep in real time with seconds’ temporal resolution and high accuracy. Our study provides evidence that the brain’s phenomenon of falling asleep displays a bifurcation dynamic and that the progression into sleep can be predicted in real time.

## Results

### Feature space trajectory of the falling asleep phenomenon: concept and implementation

The proposed framework represents the changes in the brain’s EEG activity from bedtime to sleep onset as a trajectory in a normalized *m*-dimensional geometrical (that is, Euclidean) space of EEG features. The EEG timeseries, with independent variable time (in seconds) and dependent variable scalp voltage (in microvolts), is transformed into a sequence of locations (dynamics) in a feature space of normalized EEG features (in units of s.d.), with a dependent feature space variable—the vector pointing from the instantaneous location relative to the location at sleep onset (Fig. 1a,b). The pointing vector norm represents the instantaneous distance to sleep. The timeseries of the sleep distance has an independent variable, time in seconds and a dependent variable with distance in s.d. units.

The transformation to the EEG feature space is performed by computing a set of features (feature base) over time, normalizing the feature values, and optionally offsetting the sleep onset’s feature values and timestamp. The feature base is a set of *m* mathematical computations that capture the EEG signal’s linear and nonlinear characteristics. Here we used a feature base consisting of a comprehensive set of classical EEG features quantifying the relative spectral band power distribution, peak band frequencies, bands’ temporal coherence and cross-frequency phase-amplitude couplings (PACs), plus the canonical timeseries characteristics (‘CATCH-22’) feature set shown to perform well in different types of real-world timeseries classification problems^34^ (for a total of 47 features). In some cases, we added sigma band power (12–16 Hz, sleep spindle frequency range), Lempel–Ziv complexity^35^ and aperiodic spectral slope^36^. See Supplementary Table 1 for the complete list of features and their descriptions. We computed the features in short epochs of 6 s with 50% overlap between epochs.

To normalize the features, we *z*-scored each feature to its mean and s.d. over the falling asleep period. This step converts all the EEG feature units into a comparable meaningful unit (s.d.), regardless of their physical or mathematical specifics, allowing the definition of a geometrical feature space. Using a standard score, we also mitigated a possible bias toward EEG features with larger values. We subtracted the medians over the first 10 min after sleep onset (the first continuous minute of stage N2 sleep) from the feature values to translate the sleep onset centroid to the origin of the feature space. Furthermore, we set the time at the beginning of sleep onset as time zero. These two steps facilitate easier interpretation, but they may not be essential.

The falling asleep trajectory in the feature space is described by a state vector $\bar{\bf{x}}(t)$ between the instantaneous location in the feature space and the sleep onset centroid at the origin. The norm of the state vector $\left|\bar{\bf{x}}(t)\right|$ is the instantaneous Euclidean distance to the target sleep onset centroid, which we call the ‘sleep distance’ $s\left(t\right)$, where $s\left(t\right)\equiv \left|\bar{\bf{x}}(t)\right|$. The time derivative of the state vector is the state velocity vector $\dot{\bar{\bf{x}}}\left(t\right)=\Delta \bar{\bf{x}}(t)/\triangle t$. The component of the state velocity vector toward the target sleep onset centroid is equal to the time derivative of the sleep distance—a variable we call the ‘sleep velocity’ $\dot{s}\left(t\right)=\dot{\bar{\bf{x}}}\left(t\right)\bullet \hat{x}\left(t\right)=\triangle s(t)/\triangle t$.

### Falling asleep shows a fold-bifurcation trajectory in the EEG feature space

To test the utility of the analysis approach in uncovering the brain’s falling asleep dynamics, we applied it to a large dataset of sleep EEG (*n* = 1,011, 524 women, age 69.4 ± 9.07 years, mean ± s.d.)^37,38^ with a selected range of sleep latency (minimum: 3 min, maximum: 90 min; 48.83 ± 25.01 mean ± s.d. min; median 49.5 min, interquartile range 42.38 min); see Supplementary Fig. 2 for the participants’ age and sleep latency distributions.

We first asked whether the sleep onset centroid (trajectory end) and the bedtime centroid (trajectory start) are separated in the feature space. We found that the cohort’s mean feature centroids at bedtime and sleep onset were 5.83 s.d. units (Euclidean distance) apart. The feature values at bedtime and sleep onset formed distinct clusters when the space was flattened into a two-dimensional representation using the *t*-distributed stochastic neighbor embedding approach (*t*-SNE)^39^ (Supplementary Fig. 3a). At bedtime, the participants’ coordinates (in the original high-dimensional space) were located closer to the group mean bedtime centroid than the mean sleep-onset centroid (Euclidean distance to mean bedtime centroid: 4.05 ± 1.77 mean ± s.d.; Euclidean distance to mean sleep-onset centroid: 7.11 ± 1.73; *P* < 0.00001, two-sided two-sample *t*-test). In contrast, at sleep onset, the participants’ coordinates were closer to the group mean sleep-onset centroid than the mean bedtime centroid (Euclidean distance to mean bedtime centroid: 6.82 ± 2.59; Euclidean distance to mean sleep-onset centroid: 3.86 ± 2.07; *P* < 0.00001, two-sided two-sample *t*-test). The relative distance between bedtime and sleep onset for each participant was 7.44 ± 2.64 (Supplementary Fig. 3b). These results demonstrate that the beginning and end of the falling asleep process yield distinct regions in the EEG feature space.

**Fig. 2:**
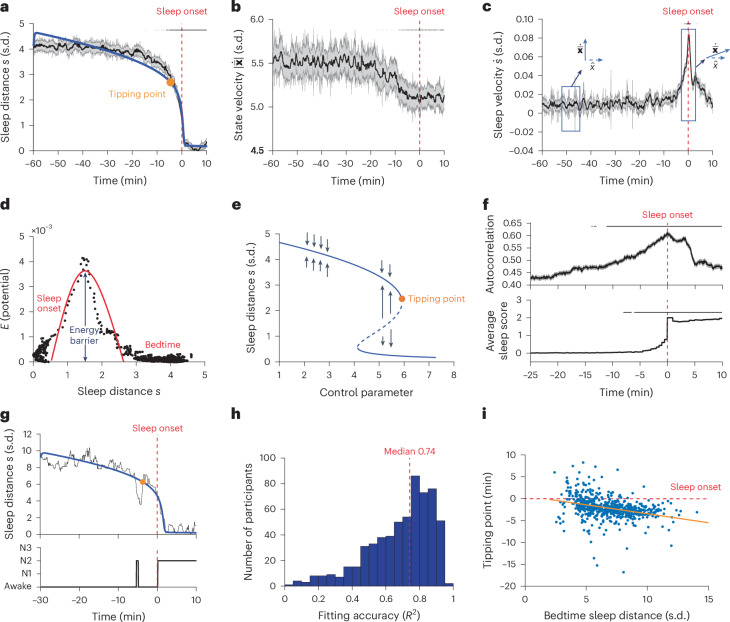
Fold bifurcation of EEG dynamics during falling asleep transition. **a**, Group-level sleep distance $s(t)$ trace. Shown are mean ± s.e.m. values per timestamp (*n* = 200). Top black lines: significant decrease relative to the first 10 min, left-tailed two-sample *t*-test with Bonferroni correction for the number of timestamps. Red dashed line: sleep-onset time throughout the figure; blue line: bifurcation function fitting; orange marker: tipping point of the bifurcation (see later). **b**, Group-level state velocity $\left|\dot{\bar{\bf{x}}}(t)\right|$ trace. Top black lines: as in **a** but using two-sided *t*-test. **c**, Group-level sleep velocity $\dot{s}(t)$ trace. Boxes highlight quasi-orthogonal vectors (before transition) and aligned states (around transition). Top black lines: as in **a** but using right-tailed *t*-test. **d**, Kinetic energy (${E}_{s}\propto {(\dot{s})}^{2}$) as a function of $s$. Red curve illustrates the potential wall between the states. **e**, Bifurcation diagram of the function fit in **a**. Solid (dashed) line segments: stable (unstable) system states attractors; black arrow: attraction directions; orange marker: tipping point of the fold bifurcation. After the tipping point, the stable system attractor (upper solid line) disappears where the system transitions catastrophically to the lower stable state. **f**, Critical slowing down of $s(t)$. Shown are autocorrelation (top) and average sleep score traces (bottom). Top black lines: as in **c** but for changes relative to first 3 min. **g**, Representative individual-level $s(t)$ trace (black) and function fit (blue) with the corresponding hypnogram (bottom). **h**, Distribution of individual-level function-fit accuracies (*R*^2^). Red dashed line: median. **i**, Tipping point time (relative to sleep onset time, dashed horizontal red line, time zero) as a function of the sleep distance at bedtime (estimated from bifurcation model fitting); orange line shows the linear regression, *r* = −0.32, *P* < 0.00001, two-sided Pearson correlation test.

Next we examined the feature space trajectory from the beginning to the end of the falling asleep process. We found that the sleep distance $s\left(t\right)$ remained relatively stable up to around 10 min before sleep onset but dropped abruptly in the last few minutes (Fig. 2a). The abrupt change in the sleep distance was not evident in the magnitude of the state velocity $\left|\dot{\bar{\bf{x}}}\left(t\right)\right|$, which showed only a slight (<10%) monotonic decrease (that is, slowing down) starting from about 15 min before sleep onset (Fig. 2b). However, it corresponded to a change in the direction of the state velocity toward the sleep onset centroid (Fig. 2c) that was associated with a peak in sleep velocity ${\dot{s}}_{\max }$. Conceptually, the transition from the wakefulness brain state into the sleep brain state had an activation energy barrier ${E}_{s}\propto {({\dot{s}}_{\max })}^{2}$ that was crossed at a distance of ~1.5 s.d. from sleep onset (Fig. 2d). An activation energy is the minimum energy required to overcome the barrier that allows the transition from one steady state (or behavior) to another (for instance, the energy required for pushing a ball from one basin to another in a landscape).

The abrupt change in the sleep distance resembles a fold bifurcation (as in a fold catastrophe model) dynamic that was hypothesized to occur during the wake–sleep transition^26,31^. To explicitly test the bifurcation hypothesis, we fitted the sleep distance variable $s\left(t\right)$ to a standard function with a fold (saddle-node) bifurcation^40,41^ (see Methods for details of the bifurcation function and its interpretation). We found that the bifurcation function accurately represented the sleep distance dynamics (*R*^2^ = 0.96, root-mean-squared error (RMSE) = 0.07), as depicted by the blue line in Fig. 2a. The bifurcation diagram (retrieved from the parameters of the fitted model) of the function shown in Fig. 2e, indicates a tipping point from wakefulness to sleep at a control parameter (which potentially captures the accumulated sleep force driving the transition) value of 5.93, occurring at 4.5 min before sleep onset (participant’s sleep stage at group-level tipping point across participants: 94.12% wakefulness, 5.55% nonrapid eye movement (NREM) stage 1 (N1), 0.003% NREM stage 2 (N2), sleep stage classification according to the American Academy of Sleep Medicine (AASM) criteria^42^).

**Extended Data Fig. 1:**
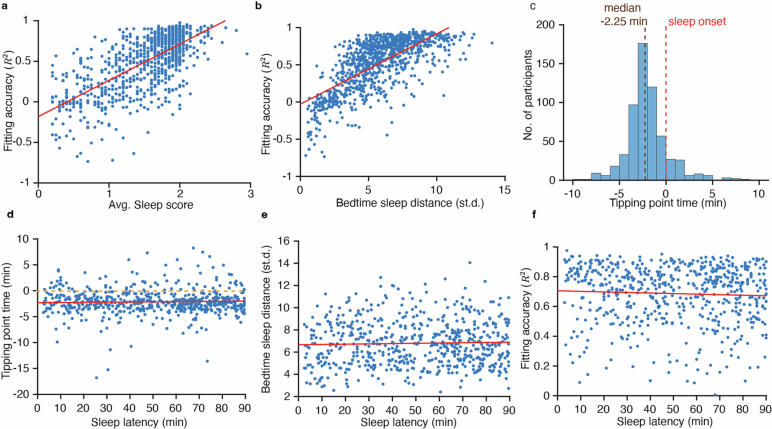
Individual-level bifurcation model fitting quantifications (related to Fig. 2). **a**, Correlation between individual-level function-fit accuracy (R-square) and average sleep score during the first 10-minutes of sleep. Red line, linear regression; *r* = 0.69, *P* = 9.41×10^−^^134^, two-sided Pearson correlation test. **b**, Correlation between individual-level function-fit accuracy (R-square) and the sleep distance at bedtime in the feature space. Red line, linear regression; *r* = 0.70, *P* = 2.26×10^−^^138^, two-sided Pearson correlation test. **c**, Distribution of the tipping point time across participants (*n* = 603); Red vertical dashed line indicates the time of sleep onset; Brown indicates the median (-2.25 min). **d**, Correlation between tipping point time (relative to sleep onset, horizontal dashed brown line, time zero) and sleep latencies. Red line, linear regression; *r* = 0.03, *P* = 0.42, two-sided Pearson correlation test. **e**, Correlation between sleep distance at bedtime and sleep latencies. Red line, linear regression; *r* = 0.03, *P* = 0.43, two-sided Pearson correlation test. **f**, Correlation between individual-level function-fit accuracy (R-square) and sleep latencies. Red line, linear regression; *r* = -0.05, *P* = 0.23, two-sided Pearson correlation test.

To test whether the bifurcation dynamics can be observed at the individual level, we fitted the bifurcation function to the wake–sleep trajectories of the individual participants. We found that the bifurcation fitting accuracy was correlated with the maintenance of sleep in the first 10 min after sleep onset, as indexed by the participant’s mean sleep score during these 10 min (Wake=0, N1 = 1, N2 = 2, N3 = 3; two-sided Pearson correlation *r* = 0.69, *P* < 0.00001; Extended Data Fig. 1a), and with the sleep distance at bedtime (two-sided Pearson correlation *r* = 0.43, *P* < 0.00001; Extended Data Fig. 1b). Both factors indicate that the accuracy of fitting depends on the distinguishability of the two states in the feature space. Figure 2g shows the sleep distance dynamics of a representative participant (*R*^2^ of fit 0.93), and Fig. 2h shows the distribution of fitting R-squared across successfully fitted (Methods) participants with consistent sleep in the first 10 min after sleep onset, that is, mean sleep score >1.5 (*R*^2^ = 0.69 ± 0.19 mean ± s.d., *n* = 603). The bifurcation accuracy was not correlated with age (two-sided Pearson correlation *r* = 0.05, *P* = 0.22).

Extended Data Fig. 1c shows the distribution of the tipping point time across successfully fitted participants (−2.15 ± 2.56 min mean ± s.d., median −2.25 min). The sleep tipping point occurred during a wakefulness stage in 70.98% of the participants, stage N1 in 15.59% and stage N2 in 13.43%. We found that the participants’ sleep tipping point time was inversely proportional to their sleep distances at bedtime, that is, a larger bedtime–sleep distance was associated with an earlier sleep tipping point (two-sided Pearson correlation *r* = −0.32, *P* < 0.00001; Fig. 2i). In contrast, neither the sleep tipping point time nor the bedtime–sleep distance was correlated with sleep latency (two-sided Pearson correlation *r* = 0.03 and 0.03, *P* = 0.42 and 0.43, respectively, Extended Data Fig. 1d,e). The sleep tipping point was correlated with age (two-sided Pearson correlation *r* = −0.10, *P* = 0.01). As in typical dynamical systems driven into a catastrophic bifurcation (such as a fold bifurcation)^33^, the individual sleep distance $s\left(t\right)$ showed a critical slowing down before the bifurcation, evident in a higher autocorrelation (Fig. 2f upper panel, coefficient 0.006, ${F}_{(1,{2.52\times 10}^{5})}=268.84$, *P* < 0.00001; linear mixed effects model (LMM)) and variance (Supplementary Fig. 4a, coefficient 0.008, ${F}_{(1,{3.52\times 10}^{5})}=31.85$, *P* < 0.00001, LMM). The increase in autocorrelation preceded the rise in sleep stage score by approximately 4 min (Fig. 2f, lower panel).

**Extended Data Fig. 2:**
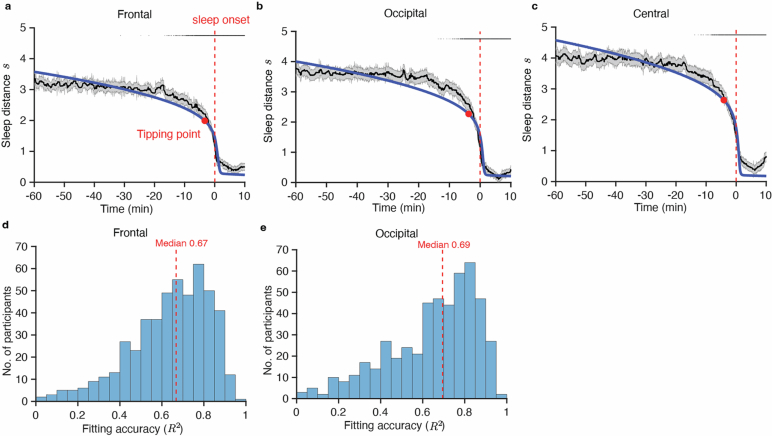
*Regional sleep distance dynamics (related to* Fig. 2*).* **a**, Same as Fig. 2a, but the sleep distance $s(t)$ is now evaluated only in the frontal region, that is, electrode derivation Fz-Cz. Model fit: R-squared 0.94, root-mean-squared error 0.05. Red circle marker, sleep tipping point, here 3.3 min before sleep onset. **b**, Same as **a** but for the occipital region, that is, electrode derivation Cz-Oz. Model fit: R-squared 0.94, root-mean-squared error 0.07. Tipping point 3.75 min before sleep onset. **c**, Same as **a** but for the central region, that is, electrode derivation C4-M1. Model fit: R-squared 0.94, root-mean-squared error 0.09. Tipping point 4.10 min before sleep onset. **d**, Same as Fig. 2h, but for the $s(t)$ dynamics in the Frontal region only (n = 496); Red dashed line shows the median. **e**, Same as Fig. 2h, but for the $s(t)$ dynamics in the Occipital region only (n = 496);; Red dashed line shows the median.

We repeated the analysis separately in the frontal, occipital and central brain regions to determine whether the observed bifurcation dynamic is conserved across the brain. We observed similar bifurcation dynamics in the sleep trajectories of each of the three brain regions (Extended Data Fig. 2a–c). The bifurcation tipping point in the occipital region preceded that in the frontal region (frontal: −3.30 min, occipital: −3.75 min). The earlier tipping point in the occipital region was associated with a larger sleep distance at bedtime (frontal: 3.76 s.d., occipital: 4.20 s.d.), consistent with Fig. 2i. The regional bifurcation dynamic was also observable in the traces of individual participants (frontal: *R*^2^ 0.64 ± 0.19 mean ± s.d.; occipital 0.65 ± 0.21; two-sided two-sample *t*-test *P* = 0.37 between the two regions; Extended Data Fig. 2d,e, *n* = 496). Again, the bifurcation tipping point in the occipital region occurred earlier than in the frontal region (frontal: −1.35 ± 3.35 min, median −1.6 min; occipital: −2.25 ± 3.19 min, median −2.38 min; *P* = 1.51 × 10^−5^, two-sided two-sample *t*-test) and was associated with longer sleep distance at bedtime (frontal: 6.02 ± 1.92 s.d., occipital: 7.79 ± 2.41; *P* < 0.00001, two-sided two-sample *t*-test), exerting an inverse relationship between the tipping point time and bedtime–sleep distance (two-sided Pearson correlation *r* = −0.36 and −0.38, for frontal and occipital, respectively, *P* < 0.00001).

**Extended Data Fig. 3:**
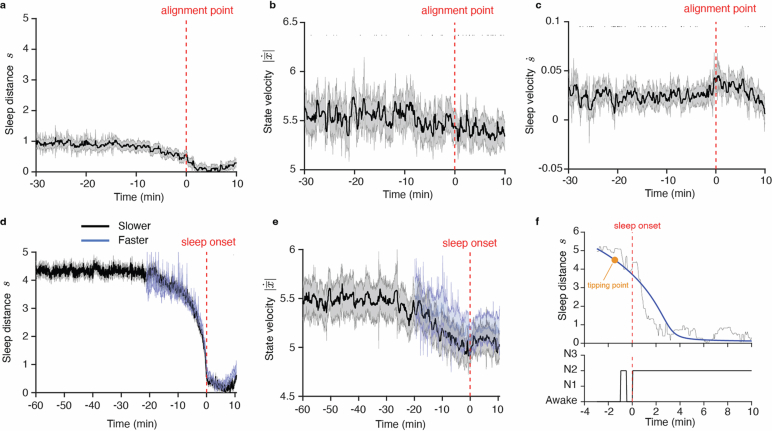
*Bifurcation confounding analysis (related to* Fig. 2*).* **a**, Group-level sleep distance $s(t)$ trace, same as Fig. 2a, but with the sleep distance $s$ evaluated when sleep onset is artificially defined at half the sleep latency of each participant. the red vertical indicates the alignment point (that is, artificial sleep onset time). **b**, Group-level state velocity $\left|\dot{\bar{\bf{x}}}(t)\right|$ trace, same as Fig. 2b but now evaluated relative to an artificially defined sleep onset as in **a**. **c**, Group-level sleep velocity $\dot{s}(t)$ trace, Same as Fig. 2c but now evaluated relative to an artificially defined sleep onset as in **a**. **d**, Comparison of the sleep distance $s(t)$ dynamics between fast (blue) and slow (black) sleepers (35% quantile split). Shown are mean ± s.e.m values per timestamp across *n* = 150 values. There was no significant difference between groups assessed via a two-sided two-sample t-test at each timestamp, with Bonferroni correction for the number of timestamps. **e**, Comparison of the state velocity $\left|\dot{\bar{\bf{x}}}(t)\right|$ between faster and slower sleepers as in **d**. There was no significant difference between groups assessed via a two-sided two-sample t-test at each timestamp, with Bonferroni correction for the number of timestamps. **f**, Same as Fig. 2g but for the representative dynamic of a participant with very short sleep latency (3 min).

To ensure that the definition of sleep onset did not artefactually cause the observed bifurcation dynamic, we repeated the analysis, but now with the sleep onset artificially defined at half the sleep latency. In this case, we did not observe bifurcation dynamics in the sleep distance (Extended Data Fig. 3a–c). We conducted similar control testing for the critical slowing down dynamics on nonfalling-asleep periods, where no significant increase in either index was observed (Supplementary Fig. 4b). To examine whether the bifurcation dynamics depended on sleep latency, we compared the sleep trajectories of slow and fast sleepers (35% sleep latency split; slower sleepers: latency >69 min; fast sleepers: latency <42 min). We did not find a difference in the bifurcation of the sleep distance (Extended Data Fig. 3d,e). See Extended Data Fig. 3f for a representative participant with a short sleep latency of 3 min. In addition, the bifurcation fitting accuracy at the individual level was not correlated with sleep latency (two-sided Pearson correlation *r* = −0.05, *P* = 0.23; Extended Data Fig. 1f).

Together, these results demonstrate that describing the brain’s falling asleep process as a trajectory in the EEG feature space unveils a robust bifurcation dynamic with a distinct wake–sleep tipping point and a phenotypical critical slowing down that precedes it.

### EEG features governing the falling asleep bifurcation

**Fig. 3:**
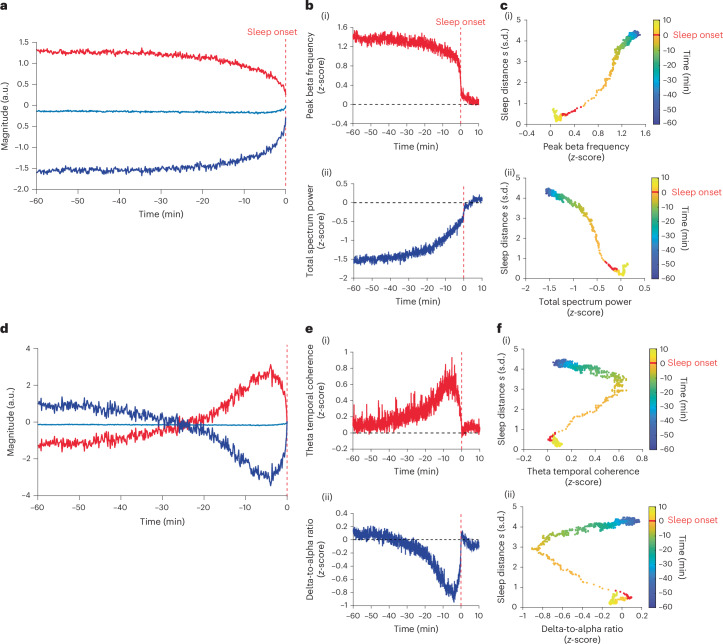
EEG features underpinning the falling asleep bifurcation dynamic. **a**, FPC1. Shown are positive (red trace, FPC1^+^) and negative (dark blue trace, FPC1^−^) reflections of FPC1 relative to the average trace of all features (light blue). Vertical red dashed line, sleep onset time; *y* axis has arbitrary units. **b**, Selected FPC1 representing EEG features. (i) Peak beta frequency feature trace representing FPC1^+^. (ii) Total spectral power feature trace representing FPC1^−^. The feature values were *z*-score normalized to sleep onset centroids (origin); vertical red dashed line indicates sleep onset time; horizontal black dashed line indicates zero *z*-score value. **c**, Sleep distance $s(t)$ trace plotted against the FPC1 representative features in **b**. Color bar indicates the time from sleep onset, with the sleep onset minute marked in red. **d**–**f**, Same as **a**–**c** but for FPC2, with delta-to-alpha ratio and theta temporal coherence feature selected to represent FPC2^+^ and FPC2^−^, respectively.

**Extended Data Fig. 4:**
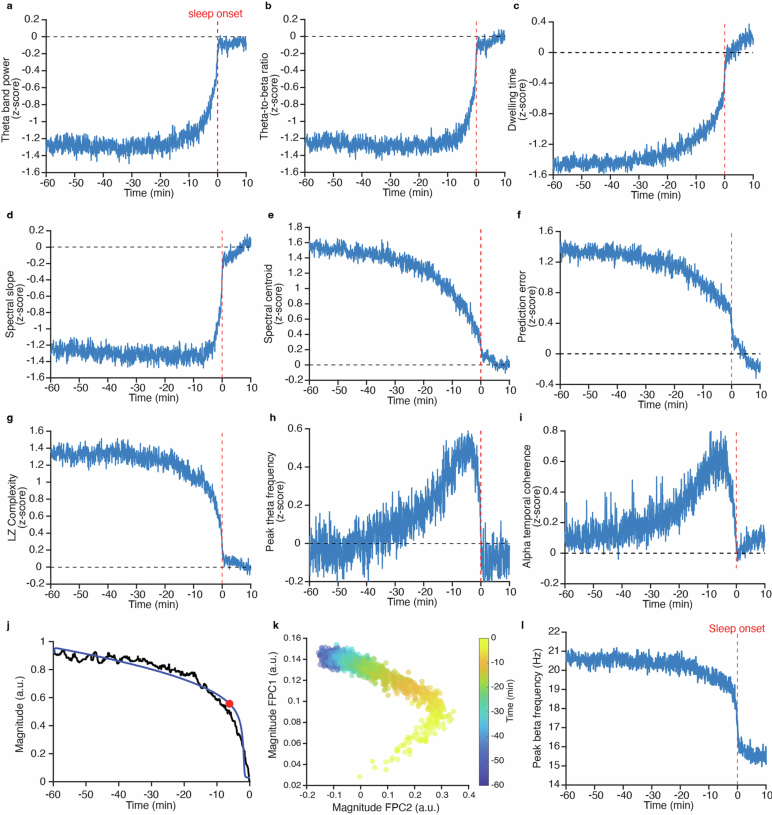
*Representative FPC features and the FPC dynamics (related to* Fig. 3*).* **a-g**, The dynamics of other representative FPC1 features (as in Fig. 3b). **h-i**, The dynamics of other representative FPC2 features (as in Fig. 3e). **j**, Group-level sleep distance $s(t)$ trace, same as Fig. 2a, but the bifurcation function was fitted on the FPC1 dynamics (black). Blue line shows the bifurcation function fit (R-squared 0.94). Red solid marker: tipping point at -5.8 min before sleep onset. **k**, FPC1 dynamics plotted against the FPC2 dynamics; Color bar shows the time. **l**, The dynamics of the peak beta frequency feature before normalisation (as in Fig. 3b).

To test whether there are EEG features that govern the falling asleep dynamic, we performed a functional principal component analysis (FPCA)^43^ of the features’ timeseries. We found that the first functional principal component (FPC1) of the features accounted for 96% of the total variance (Fig. 3a). The timeseries of FPC1 displayed bifurcation dynamics (*R*^2^ = 0.94; Extended Data Fig. 3j). From the feature scores, we found that the positive FPC1 was best represented by the peak beta frequency, spectrum centroid, Lempel–Ziv complexity and prediction error features (maximum score = 11.22, normalized to the maximum scores: 1, 0.99, 0.95 and 0.92, respectively; reporting all features with normalized score >0.9). The peak beta frequency drops from ~21 Hz (above the spindle range) to ~15.5 Hz (close to the upper frequency range of sleep spindles) at the bifurcation (Extended Data Fig. 4l). The negative FPC1 was best represented by the dwelling time, total spectrum power, spectral slope, theta band power and theta-to-beta band power ratio features (minimum score = −9.10, normalized to the maximum scores: −1, −0.97, −0.96, −0.92, −0.92, respectively). See Extended Data Table 1 for the list of scores for all features and Supplementary Table 2 for feature interpretations. Figure 3b shows the timeseries of the peak beta frequency and the total spectrum power features (see Extended Data Fig. 4a–g for the timeseries of the other representative features). Figure 3c shows the sleep distance $s\left(t\right)$ plotted against the FPC1 representative features, revealing a quasi linear relationship with reduced datapoints at the bifurcation (two-sided Pearson correlation: *r* = 0.99 and *r* = −0.97, respectively; *P* < 0.0001).

**Extended Data Fig. 5:**
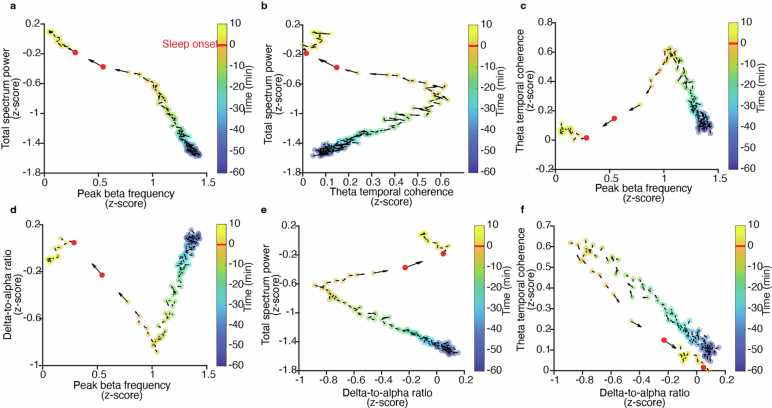
Relationship *between FPC representative features (related to* Fig. 3*).* **a-f**, 2D plots of the relationship between the four FPC representative features, that is, peak beta frequency, total spectrum power, theta temporal coherence, and delta-to-alpha ratio. Each dot represents the group median feature values in a 30-s segment, with arrows showing the median direction vector (from the data points of the current 30-s segment to those of the next), and the arrow length indicates the magnitude of the vector. The colour shows time, with the sleep onset minute marked in red.

The second functional principal component (FPC2) of the features accounted for only ~3% of the variance (Fig. 3d). The timeseries of FPC2 peaked around 4.5 min before onset, that is, at the time of the bifurcation tipping point (as in Fig. 2a). The positive FPC2 was best represented by the theta temporal coherence, alpha temporal coherence and peak theta frequency features (maximal score = 1.80, normalized to the maximum scores: 1, 0.99, 0.95). The negative FPC2 was best represented by the delta-to-alpha ratio feature (score = −2.33). Figure 3e shows the timeseries of the theta temporal coherence and the delta-to-alpha ratio features (see Extended Data Fig. 4h,i for the timeseries of the other features). Figure 3f shows the sleep distance $s\left(t\right)$ plotted against the FPC2 representative features, showing a phenotypical fold bifurcation resembling the control parameter (a parameter in which a gradual change causes system bifurcation). See Extended Data Fig. 4k for FPC1 dynamics against FPC2. To explicitly test whether the FPC2 features could represent the bifurcation’s control parameter, we quantified the cosine similarity and dynamical time warping (DTW) distance^44^ between these features and the bifurcation control parameter of the sleep distance $s\left(t\right)$. We found that theta temporal coherence best represented the control parameter (theta temporal coherence: cosine similarity 0.94, DTW distance 6.69). See Supplementary Table 3 for cosine similarity and DTW distances of other features. See Extended Data Fig. 5 for the relative changes between the representative features in Fig. 3.

**Extended Data Fig. 6:**
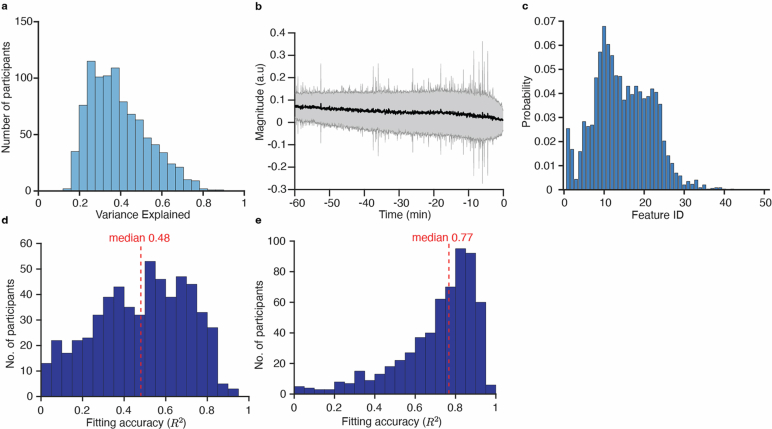
*Individual-level FPCA analysis (related to* Fig. 3*).* **a**, Distribution of the variance explained by the first functional principal component (FPC1) on the individual level (*n* = 940 in total). **b**, Group-averaged dynamics of individual FPC1 traces; Shown are mean ± standard error of the mean (s.e.m) values per timestamp (*n* = 200). **c**, Probability of each feature being FPC1 representative features (normalised score larger than 0.9 or smaller than -0.9) across all individuals. The horizontal axis corresponds to the feature indexes (50 features in total, in the same order as Extended Data Table 1). **d**, Same as Fig. 2h but for R-squared distribution of function fitting for sleep distances evaluated with only one feature dimension (the peak beta frequency). **e**, Same as Fig. 2h but for R-squared distribution of function fitting for sleep distances evaluated in the feature space consisting of all nine FPC1 representative features.

The bifurcation in the group-level FPC1 and the fact that it can be accurately represented by individual features, such as peak beta frequency, raised the question of whether these features can effectively capture falling asleep bifurcation dynamics independently (that is, without a feature space). To address this question, we fitted the bifurcation function to the participant’s FPC1 representative features timeseries (absolute values). We found that the timeseries of the individual features did not consistently display bifurcation dynamics, as evident in a lower fitting accuracy than sleep distance (Extended Data Table 2). For example, the peak beta frequency with the highest FPC1 score had an *R*^2^ value of only 0.43 ± 0.28 (mean ± s.d.), Extended Data Fig. 6d. In contrast, the trajectory in a feature space consisting of only the nine FPC1 representative features displayed the bifurcation in the sleep distance variable (*R*^2^ = 0.70 ± 0.21 mean ± s.d., *n* = 603; *P* = 0.99, two-sided two-sample *t*-test against *R*^2^ distribution of the complete feature space), see Extended Data Fig. 6e for *R*^2^ distribution. To test whether each participant may have a unique feature that captures the bifurcation dynamics, we repeated the FPCA of the EEG features timeseries for each participant and examined the individual FPC1s. We found that, in contrast to the group-level FPC1, the individual FPC1s captured only a small proportion of the features’ variance (39% ± 14%, mean ± s.d.; Extended Data Fig. 6a) and did not display bifurcation dynamics (Extended Data Fig. 6b). Furthermore, the representative features of FPC1 varied considerably across participants (Extended Data Fig. 6c).

Overall, the display of bifurcation in the group-level FPC1 strengthens the choice of a bifurcation model to describe the falling asleep phenomenon and indicates that a low-dimensional feature space can capture the bifurcation dynamics but not individual EEG features. The resemblance of FPC2 to the bifurcation control parameter may further support the choice of a bifurcation model. Yet, neurophysiological sleep drive inferences cannot be made without further, perhaps more direct, investigation.

### Prediction of falling asleep dynamics

**Extended Data Fig. 7:**
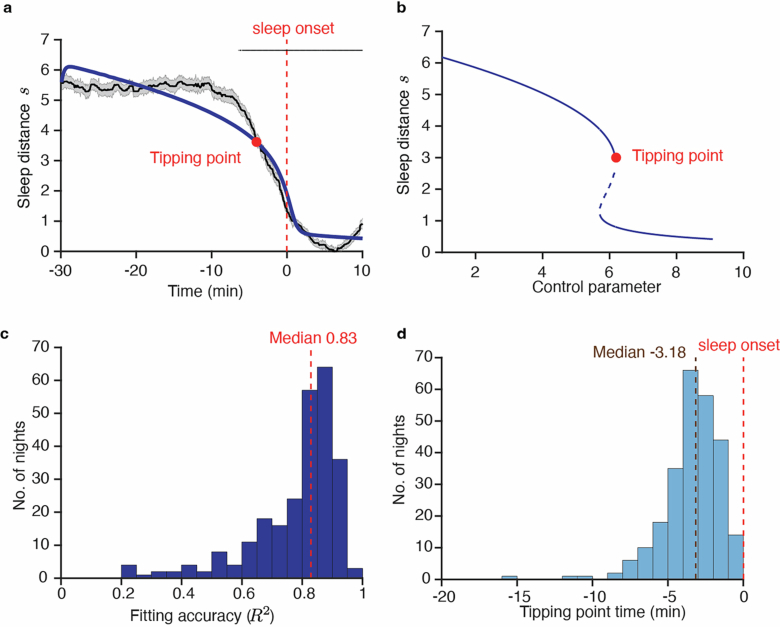
*Replication of the bifurcation dynamic in cohort 2 (related to* Fig. 4*).* **a**, Group-level sleep distance $s(t)$ trace the same as Fig. 2a but for EEG data from cohort 2; *n* = 150 participants per data sample (bootstrapped due to differences in sleep latencies). The horizontal black line at the top of the panel indicates a significant decrease relative to the first 5 min, left-tailed two-sample t-test with Bonferroni correction for the number of timestamps **b**, Bifurcation diagram of the function fitted to the group-level $s(t)$ trace (blue line in a). Red marker indicates the tipping point (-4.05 min before sleep onset). **c**, Accuracy of bifurcation representation of sleep distance $s(t)$ traces across all nights (*n* = 256), showing R-squared distribution with the vertical red dashed line showing the median (0.83). **d**, Distribution of the tipping point time across nights (*n* = 256); Red vertical dashed line indicates the time of sleep onset; Brown indicates the median (-3.18 min).

Finally, we explored whether the computational framework can be used to predict individual transitions to sleep in a second data cohort. We recorded sleep EEG of individual participants across consecutive nights (*n* = 36 participants, 18 women; age 27.42 ± 4.02 years (mean ± s.d.); 267 total nights, 7.42 ± 0.97 mean ± s.d. nights per participant; sleep latency 42.30 ± 29.25 min, median 33.5 min, interquartile range 37.63 min). See Supplementary Fig. 5 for age and sleep latency distributions (note that the feature base here did not include sigma band power, Lempel–Ziv complexity and spectral slope). Similar to the first cohort, we found a bifurcation dynamic in the sleep distance trace $s\left(t\right)$ of this cohort as the participants fell asleep (*R*^2^ = 0.95, RMSE = 0.23), Extended Data Fig. 7a,b. As before, the bifurcation dynamic was also observable in the individual recordings (mean fitting *R*^2^ across participants and nights 0.78 ± 0.15 s.d., *n* = 256 nights; Extended Data Fig. 7c; mean *R*^2^ per participant across nights 0.78 ± 0.10 s.d., *n* = 36 participants). The bifurcation fitting accuracies in this cohort were slightly better than those of the first cohort (*P* < 0.00001, Kruskal–Wallis test). Again, the bifurcation fitting accuracy was correlated with the maintenance of sleep in the first 10 min after sleep onset (two-sided Pearson correlation *r* = 0.32, *P* < 0.00001) and with the sleep distance at bedtime (two-sided Pearson correlation *r* = 0.48, *P* < 0.00001). The bifurcation fitting accuracy was correlated negatively with sleep latency (two-sided Pearson correlation *r* = −0.25, *P* = 7.15 × 10^−5^). Extended Data Fig. 7d shows the distribution of the tipping point time across participants (tipping point time across participants and nights −3.40 ± 1.94 min mean ± s.d., median −3.18 min; mean tipping point time per participant across nights −3.37 ± 1.19 s.d.). Eleven nights showed invalid tipping points (see Methods for details). As before, the sleep tipping point time was inversely proportional to the sleep distance at bedtime (two-sided Pearson correlation *r* = −0.32, *P* < 0.00001) but not to sleep latency (two-sided Pearson correlation *r* = 0.06, *P* = 0.34). The sleep tipping point occurred during wakefulness stage in 30.98% of the nights, stage N1 in 68.63%, and stage N2 in 0.003% (sleep stages classified according to Rechtschaffen and Kales (R&K) criteria^45^ but mapped to AASM; Methods).

To explore the feasibility of individual prediction, we first tested whether the participants’ sleep onset locations (that is, the median coordinates across the first 10 min of sleep) were consistent across nights. We found that, for 83% of the participants (30 out of 36 participants), the sleep onset locations across nights could not be separated by clustering (mean Silhouette score 0.03 ± 0.09 across all participants, significance compared to 1,000 surrogates per participant, *P* value threshold 0.05). See Extended Data Table 3 for the Silhouette scores and *P* values of each participant. See Supplementary Fig. 6 for a two-dimensional view of sleep onset centroids in the feature space. In contrast, the median values (across the first 10 min of sleep) of the features representing FPC1 varied significantly across nights (Supplementary Table 4).

**Fig. 4:**
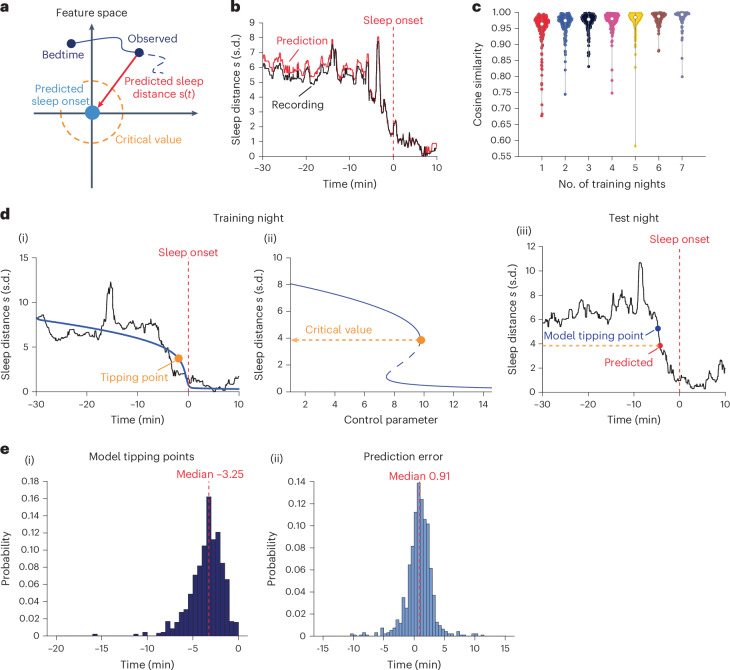
Prediction of individual sleep bifurcation dynamic. **a**, Sleep dynamic prediction in feature space. Origin (light blue): the predicted sleep-onset centroid from training night. The sleep distance $s(t)$ traces of testing nights could be predicted by evaluating the Euclidean distance toward the origin (red arrow). Dashed orange curve: the critical value derived from the training night (see **d**). **b**,**c**, Prediction of $s(t)$. Representative night showing the $s\left(t\right)$ trace in black (recording) and red (prediction, cosine similarity 0.99) (**b**). Red dashed line: sleep-onset time throughout the figure. Cosine similarity (accuracy) across all test nights versus the number of training nights used (**c**). Violin plot show the kernel density (shape), median (nonfilled circle), and upper and lower 25% quantiles (gray bar). **d**,**e**, Prediction of tipping points. Representative participant (**d**). ((i)–(ii)) Training night, showing (i) $s(t)$ trace in black and in blue the fitted bifurcation function (R-squared 0.79) and (ii) bifurcation diagram of the fit. Orange marker, tipping point of the bifurcation. Orange line, tipping point’s critical sleep distance. (iii) Test night, showing the predicted $s(t)$, with the post hoc model (blue) and predicted (red) tipping points, respectively. The prediction error was then defined as the time of the predicted tipping point (red) minus the one given by the post hoc model (blue). Prediction of tipping point across all test nights (**e**), showing distributions of tipping point computed post hoc (i) and tipping point prediction error (ii).

Next, we tested whether the participant’s sleep onset location from a subset of nights (training data) could be used to predict the instantaneous sleep distance traces $s\left(t\right)$ in the other nights (test data) in pseudo-real time (Fig. 4a). We were able to accurately predict the progression into sleep (that is, $s\left(t\right)$ trace) on the fly using the sleep onset location from only a single night (randomly selected, repeated five times, *n* = 180 cases; cosine similarity 0.95 ± 0.06). Adding information from more nights improved the accuracy only slightly (~0.005 per additional night). Figure 4b shows a representative prediction of sleep distance $s\left(t\right)$ trace, and Fig. 4c shows a group-level summary of the prediction accuracy versus the number of training nights (*n* = 1,132 testing cases, see Supplementary Table 5 for cosine similarity statistics).

Next, we tested whether this approach allows us to predict the participants’ wake–sleep bifurcation tipping point crossing in real time. We fitted the bifurcation function to the sleep distance trace of a single night (randomly selected, repeated five times) and found the critical sleep distance at which the tipping point occurred. We then pinpointed when the instantaneous sleep distance crossed the predicted critical value on other nights in pseudo-real time and compared it to the tipping point computed using post hoc fitting of the bifurcation function. We found that using the bifurcation model of a single night, we were able to predict the tipping point crossings of the other nights with a mean time error of 0.82 ± 2.14 min relative to post hoc fitting of the bifurcation function (*P* < 0.0001, one-sample *t*-test for the prediction error; *n* = 944 testing nights; 141 nights, that is, 13%, showed invalid predicted tipping point, see Methods for details). Figure 4d shows a representative result, and Fig. 4e(i),(ii) show the distributions of the tipping point time (−3.53 ± 1.80 min) and prediction error, respectively. In a subset of nights (0.32 ± 0.67 out of 7.42 mean ± s.d. nights per participant), the sleep distance $s\left(t\right)$ trace crossed the critical value more than once, leading to an erroneous early tipping point crossing prediction. Refer to Supplementary Fig. 7a for an example and Supplementary Fig. 7b for the distribution of these instances. These occurrences were potentially linked to unsuccessful sleep transitions associated with a shift into the N1 or N2 stage. The likelihood of such an erroneous early tipping point prediction was associated with a higher fluctuation in the hypnogram (that is, the number of sleep stage transitions before sleep onset; two-sided Pearson correlation coefficient *r* = 0.24; *P* = 0.001; Supplementary Fig. 7c).

## Discussion

This paper presents a computational framework to analyze and model the phenomenon of falling asleep. It then uses it to demonstrate that changes in brain activity display a bifurcation dynamic that is conserved within each individual person and can be predicted in real time. The computational framework represents the changes in brain EEG activity as a trajectory in a normalized feature space relative to sleep onset (descriptive metric). We used a feature base consisting of a comprehensive set of classical EEG features and a set of features optimized for timeseries data^34^. The feature base was proven efficient for a clear separation of the two states to capture the dynamics of the wake–sleep transition, but it is not exclusive. For example, it is plausible that adding features capturing intersite linear and nonlinear correlations and/or causation^46^ will improve the representation. In addition, our epoching choice is not unique or systematically optimized. We *z*-score normalized each feature to its mean and s.d. over the falling asleep period. The normalization converts all the EEG feature units into comparable, meaningful units. The *z*-score normalization, that is, standard score, used in this paper has proven efficient but is not unique. It is plausible that other normalization approaches would also work well. We defined the sleep onset centroid as the median over the first 10 min after sleep onset (the first continuous minute of stage N2 sleep) instead of the sleep onset minute as a better representation of the new sleep onset state, rather than just the beginning of the new state. It also avoids an artefactual sleep distance extremum at that single point (for example, sleep onset minute). However, this definition of sleep onset centroid is not exclusive or systematically optimized. The transformation results in an *m*-dimensional Euclidean state space representation of the brain EEG changes. The state space is not canonical as it consists of correlated state variables (EEG features). We show the feasibility of deriving a low-dimensional space using a popular dimensionality reduction technique (FPCA). Note that our state space representation does not assume specific attractors or a particular transfer function. Representing the brain’s progression into sleep as a trajectory in such a feature space is a simple and intuitive descriptive quantification of the falling asleep phenomenon that accentuates the sleep-relevant physiological changes while being agnostic to the specifics of the individual governing features.

We show that fold-bifurcation models can accurately describe the sleep distance trace during the transition into sleep, independent of sleep latency and age. The existence of a phenotypic critical slowing down before the bifurcation and earlier theoretical computational model studies^26,28,31^ support the choice of a bifurcation model. Nevertheless, we did not test the bifurcation model against alternative models and, hence, it is plausible that other dynamical systems models can also represent the falling asleep process. We used a standard fold bifurcation function^40,41^ to model the sleep distance trace, but it is plausible that other functions with similar bifurcation properties might also work. The accuracy of the model fitting was worse at early timepoints before the bifurcation, potentially due to a higher sensitivity to sleep-irrelevant brain dynamic changes or a more subtle dynamic related to the dominance of subcortical drive^47^ and/or dispersed cortical regulation^48^. The bifurcation function and fitting could be optimized in the future to better represent the dynamics of this period and potentially reveal the abnormalities in those with sleep disorders. We focused on ‘normal’ sleep onset with latencies longer than 3 min and at ‘normal’ sleep time. Yet, future studies could assess the model’s performance in much shorter sleep latencies, for example, after sleep deprivation, in patients with narcolepsy, and sleep onset following awakening. Future studies could also investigate the process by which sleep is initiated at other times of the day or circadian cycles. The wake–sleep bifurcation model requires a clear delineation of stable wake and sleep states, which might be challenging in conditions with less-defined transitions or mixed states.

Embedding the feature space trajectory descriptive model in the mathematical bifurcation model grounds the computational framework in the rich theory of bifurcations^49^. This provides experimental evidence that the brain’s falling asleep phenomenon displays a bifurcation dynamic (see Supplementary Notes for a summary of earlier attempts). Bifurcation theory enables us to compute a sleep tipping point, that is, the moment at which sleep distance is reduced below a critical value that marks an accelerated point-of-no-return transition from a wakefulness steady state into a sleep steady state. The definition of sleep onset latency, that is, the wake–sleep cutoff point, is essential for diagnosing and managing several sleep disorders, such as insomnia, narcolepsy and excessive daytime sleepiness^5^. The traditional sleep onset definition is delimited as the first 30 s scored as N1 or N2, or the first two to three consecutive epochs labeled sleep^5,50^. However, the arbitrary nature, coarse timestep, and low interscorer agreement at the early stages of sleep have limited the utility of this definition^51^. The sleep bifurcation tipping point does not align with traditional sleep stages and can occur during wakefulness or sleep annotations, thereby challenging the conventional definition of wakefulness and sleep. The tipping point accurately defines the critical time during the transition from a steady wake state into a steady sleep state, providing an objective and physiologically precise definition of the wake–sleep cutoff. The bifurcation theory also postulates that a system driven toward the tipping point becomes disturbed more easily, reflected in larger variance and autocorrelation—a phenomenon known as critical slowing down^33^. Our results show a significant increase in the variance and autocorrelation of the sleep distance timeseries minutes before the sleep tipping point. The critical slowing down in the sleep distance trace can be used to predict upcoming sleep tipping points^52^. Lastly, the framework helps generalize distinct EEG feature dynamics with potentially different roles underpinning the bifurcation process. Future studies can utilize these findings to discover more mechanistic interpretations of these features.

Our findings suggested that an earlier sleep bifurcation tipping point is associated with larger sleep distance at bedtime, which may seem counterintuitive. In addition, we show that the occipital brain region displays an earlier sleep bifurcation tipping point and a larger bedtime–sleep distance at bedtime than the frontal region, which seems to contrast earlier reports of a fronto-posterior falling asleep gradient when monitoring the low-frequency band power^6,13,53^. However, a larger sleep distance at bedtime reflects a brain state further from sleep, but not necessarily a brain state closer to arousal, as it is a multidimensional space. In addition, an earlier bifurcation tipping point reflects a longer bifurcation transition process but not shorter sleep latency. The dependency of the bifurcation tipping point time on the sleep distance at bedtime might suggest a distance-independent acceleration (and hence attraction force) during the bifurcation—larger distance takes longer time and therefore is associated with an earlier bifurcation onset. The wake–sleep bifurcation kinematics resembles the kinematics of a falling body, which is characterized by a constant acceleration and a falling time that depends on the initial distance, thereby supporting the subjective sensation of ‘falling’ asleep. This type of kinematics does not resemble the kinematics of a spring-mass system that was proposed recently^54^—a spring-mass is characterized by a displacement-dependent acceleration—but a time to relaxed spring position that is independent of the initial displacement. Future studies might be able to better pinpoint the dependency of the bifurcation tipping point on the bedtime–sleep distance (and hence the attraction force) by exploring nonlinear relationships. Sleep onset disturbances are more common in older adults^55^ and are associated with an increased risk of Alzheimer’s disease^56^. Our findings suggest that the wake–sleep bifurcation dynamic is preserved with age, yet with an age-dependent tipping point. The fitting accuracy in the second cohort (younger participants) was slightly better than in the first cohort (older participants), but this could be attributed to factors other than age (for example, EEG montage and recording quality). Future studies should explore whether and how specific bifurcation characteristics change with age.

The wake–sleep computational framework we proposed integrates a feature space trajectory descriptive model and a dynamical systems bifurcation model. To our knowledge, the proposed framework is new. We note the report by Diniz Behn et al. on mouse wake, NREM and REM sleep stages, showing distinct clusters in a two-dimensional state of low and high power-band ratios^57^. Hitherto, the characterization of the transition into sleep has been based on EEG patterns or specific EEG features, such as Hori’s nine-stage system^9^. Similar to standard sleep scoring, this staging system also suffers from large inter-rater variability and is time consuming^5^. More recent analyses based on quantitative EEG analysis, such as power spectrum properties^6,58^, nonlinear dynamics and connectivity^59^, or spatial topographies as microstates^18,20,21^ are constrained by individual differences in the EEG changes underpinning the falling asleep phenomenon, the frequent need for hyperparameter tuning, and the lack of prediction capabilities. In our framework, the changes in the EEG are transformed into a trajectory in a normalized feature space. We mitigate bias in features with larger amplitudes by standardizing the feature values. We reduced sensitivity to intersubject variability in the governing features by measuring a global quantity, that is, a Euclidean distance. We reduce sensitivity to sleep-irrelevant changes in brain activity by expressing the distance relative to the target sleep onset centroid.

Hitherto, frameworks to model the wake–sleep transition phenomenon have been based on an abstraction of the neural processes or circuits that regulate sleep. In the classical two-process model^25^, sleep propensity is modeled as a sum of two high-level oscillating processes: a sleep homeostasis process S that depends on sleep–wake history and a circadian process C independent of history. The onset and termination of sleep are defined as H and L thresholds, respectively, that depend on the circadian rhythm (process C). Physiologically, it has been shown that S can be estimated from EEG slow-wave activity^60^, and C can be estimated from body temperature and plasma/salivary melatonin levels^61^. In contrast, the neural circuit models directly describe interactions between relevant neural populations. A classic model by McCarley et al.^62^ implemented the reciprocal interaction between REM and NREM regulation systems as a competitive Lotka–Volterra equation. The wake–sleep transitions are governed by a mutual inhibition interaction between wake-promoting (ascending reticular arousing systems) and sleep-promoting (mainly the hypothalamic ventrolateral preoptic nucleus) neural populations. Based on this, Phillips and Robinson implemented a neural mass model with a mean cell body voltage representing each population^26^ (see also refs. ^27,28^). The parameters of these two types of models are closely related^29^. Both models have monophasic and polyphasic sleep patterns with a bifurcation transition between them^30,31^. These models have been used extensively to study day-long macroscopic wake–sleep changes and transitions between different sleep stages, but less commonly the fast transition dynamics in depth, despite their capabilities to do so^63^. Yang et al. showed that the governing mathematics of the Phillips and Robinson model predicts a bifurcation dynamic near the wake–sleep transition^31^, a prediction we validated experimentally in this paper. Overall, the computational framework described in this paper is distinct from these physiological abstractions of sleep regulation. Specifically, our framework is a nonphysiological abstraction of the physiological changes in brain EEG activity during the wake–sleep state transition. Future studies should explore whether the proposed framework can help uncover the brain dynamics during other brain state transitions, such as sleep to wakefulness. A consistent saddle-node bifurcation dynamic in the brain’s wake–sleep and sleep–wake transitions would predict hysteresis, that is, an awakening centroid distinct from the bedtime centroid and closer to the sleep onset one, thus the sleep inertia effect^64^.

The FPCA of the group average features’ timeseries suggests that most of the variance (97% in our case) can be explained by the FPC1. The timeseries of FPC1 displays a bifurcation dynamic such as the timeseries of the sleep distance in the feature space. The timeseries of a subset of features, that is, the peak beta frequency, spectrum centroid, Lempel–Ziv complexity, and prediction error, followed the positive FPC1 timeseries (values dropped abruptly at sleep onset). In contrast, the timeseries of another subset of features, that is, the dwelling time, total spectrum power, spectral slope, theta band power, and theta-to-beta band power ratio, followed the negative FPC1 timeseries (values abruptly increased at sleep onset). The abrupt increase in the classical theta band power and theta-to-beta band power ratio features is associated with the well-established emergence of slow oscillatory activity at the transition to sleep^58^. It is conceivable that the superposition of the slow (theta band) oscillatory activity and ongoing nonoscillatory aperiodic 1/*f* activity is associated with the increase in the total spectrum power feature and spectral slope feature (steeper spectrum power drops with frequency) and the decrease in the spectrum centroid (shift of spectrum center of mass to low frequencies). It is also plausible that the emergence of slower oscillatory activity is associated with the reduction in the EEG Lempel–Ziv complexity and prediction error (datapoints are more correlated) and the increase in the dwelling time (slower fluctuation of EEG values relative to their mean). The abrupt reduction in the peak beta band frequency from ~21 Hz (above the spindle range) to ~15.5 Hz (close to the upper frequency range of sleep spindles^65^) might be associated with the emergence of spindle activity. Future studies might be able to establish a quantitative relationship between the classical emergence of oscillation in sleep and changes in these features.

Our results show that the location of sleep onset in the feature space is preserved in individual people across several nights, independently of the location in the feature space at bedtime. We demonstrate how this property can be used to accurately predict the instantaneous progression into sleep after only a single night of recording. The bifurcation dynamic is also preserved in people across several nights, allowing real-time prediction of approaching the sleep tipping point, albeit with lower accuracy. These results together demonstrate the capability of real-time prediction of the brain’s progression into sleep. We note the report by Prerau et al.^66^, who derived a probability of losing behavioral response while falling asleep, quantified by the accuracy of pressing a stress ball during breath inhales. However, an active task can bias the natural phenomenon^67^. Moreover, the task performance does not accurately represent the complex changes in brain activity. See Supplementary Notes for a detailed summary of earlier attempts.

Sleep is a fundamental part of our lives; however, how our brain falls asleep has remained a mystery. This paper provides a new conceptual framework to uncover this long-lasting mystery and demonstrates a bifurcation dynamic with real-time prediction capabilities. The framework and findings may help uncover new mechanistic understandings of the falling asleep process, develop new strategies to diagnose and treat sleep onset disorders and mitigate extraneous sleep during duty or on the road.

## Methods

### Definition of sleep onset

We conservatively defined the sleep onset as the first 1 min of stage N2 sleep (two continuous N2 epochs). Many previous works have proposed stage N2 sleep as a safe boundary between wakefulness and sleep^50,68^. Comparably, stage N1 sleep is a more transient state with heterogeneous features of both wakefulness and definite sleep^69,70^ and has been shown to have the lowest inter-rater consensus^71^. By defining at least two continuous epochs of stage N2 sleep, we minimize the impact of biased sleep staging as given in the AASM criterion, that is, the dominant pattern determines the sleep stage of one specific 30-s epoch^42,72^. Still, this definition is not perfect, and the participants could wake up again after this point, which is an important factor that affects our model performance (see results). Note that we subtracted the timestamp of sleep onset to express time relative to sleep onset, where the beginning of sleep onset was time zero (for example, the red dashed line in Fig. 2a), that is to say, the two continuous Stage N2 sleep occur after our marked *t* = 0.

### EEG timeseries feature base and its computation

#### Development of the feature base

We developed a feature base to capture comprehensive characteristics of EEG dynamics for the falling asleep period. We incorporated the EEG power spectrum features (band powers, band power ratios and peak oscillation frequencies), whose dynamical changes have been explored extensively in previous works^5,6,58^. We further incorporated other EEG features that are used more commonly in other scenarios, such as cross-frequency coupling^73^, and temporal coherence (a measurement of internal oscillation pattern coherence)^74^. As timeseries data, there is a massive repertoire of analytic features outside the normal spectrum analysis (which is linear) that could provide interesting unknown insights into neural dynamics during falling asleep transition. For instance, several nonlinear dynamical metrics have been shown to classify sleep stages^75,76^. We thus added a set of 22 features called CATCH-22 (ref. ^34^), which is the best representative set out of over 7,000 timeseries features^77^ and comprises many nonlinear metrics outside the Fourier domain. In cohort 1, we also added sigma band (12–16 Hz) power, normalized Lempel–Ziv complexity (entropy rate) that was shown to reflect arousal level^78,79^, and EEG spectral slope, suggested to change during sleep^35^. In total, we used 50 EEG features in the first cohort (Figs. 2–3) and 47 features in the second (Fig. 4). See Supplementary Table 1 for the full feature list.

#### Computation of the features

The feature computation was conducted in MATLAB 2023a (Mathworks Inc.). The EEG signals were preprocessed and epoched into 6-s segments, and the features were evaluated for each epoch.

#### Band power features

The EEG was first bandpass filtered between the (0.1,32) Hz range. We then applied a differentiation filter to remove the 1/*f* spectrum component^80^ to better capture the periodic component of the signal. The power spectral density (PSD) for this epoch was then estimated using Thomson’s multitaper approach (MATLAB function pmtm), with four tapers and the number of fast Fourier transform points the same as the sampling frequency (256). The PSD was then normalized to the total spectrum power so that the band power is a ratio compared to the entire spectrum. The band powers were computed by averaging the normalized PSD of all the frequency bins within that band and then log-transformed. Similarly, the band power ratios were computed as the quotient of the band powers and then log-transformed. The peak frequency in each frequency band was defined as the frequency bin with maximum normalized power. The four frequency bands were defined as delta band 1 Hz to 4 Hz, theta band 4 Hz to 8 Hz, alpha band 8 Hz to 12 Hz and beta band 12 Hz to 30 Hz. The Sigma band power was calculated as the mean power within the 12–16 Hz band.

#### PAC features

PAC is normally measured between different EEG channels; here we turned it into a local feature. We first applied the same differentiation filter to ensure the same spectrum property. The PAC was then evaluated by the following methods^81^. Briefly, for the input EEG epoch, we designed a zero-phase, even-order finite impulse response filter to filter the signal into the four respective bands (defined as before). For each frequency band, we then applied the Hilbert transform to calculate the phase and amplitude. Phase cycles were then extracted (from −*π* to *π* is a cycle) and, for each cycle, the modulation phase and amplitude are put into a complex signal: $z\left(t\right)={A}_{f1}\left(t\right)\times {e}^{{\varphi }_{f2}(t)}$, where ${A}_{f1}$ is the amplitude of higher frequency band oscillations, and ${\varphi }_{f2}$ is the phase of lower frequency band oscillations. The PAC was then quantified by summing $z\left(t\right)$ over all phase cycles and then normalizing to the average amplitude ${A}_{f1}$ in each cycle. The result PAC value is between 0 and 1, where closer to 1 implies stronger coupling. The PAC was thus calculated for each frequency band pair (lower-band phase to higher-band amplitude only).

#### Temporal coherence features

For each EEG epoch, we first applied the same differentiation filter as for band power features. We further divided the epoch into six 1-s segments, and the temporal coherence per frequency was quantified using the magnitude squared coherence algorithm using MATLAB’s mscohere function. Briefly, the coherence spectrum was evaluated between each pair of the 1-s segments and then averaged. For each frequency band, the feature value was calculated as the sum of the values of all the frequency bins within that band. The temporal coherence features were evaluated for those four frequency bands specified before.

#### CATCH-22 features

CATCH-22 (ref. ^34^) has an openly available toolbox (https://github.com/DynamicsAndNeuralSystems/catch22, v.0.1.0) implemented in both MATLAB and Python. We used the function provided to evaluate the 22 features for each epoch directly.

#### Additional nonlinear EEG features

The Lempel–Ziv complexity (evaluated in its normalized form named Entropy rate) was calculated on each epoch using the EntRate toolbox (https://github.com/pmediano/EntRate); The EEG epoch was first binarized and Lempel–Ziv 76 complexity was evaluated; the entropy rate was calculated as ${Lz}\times \frac{{\log }_{2}l}{l}$, where ${Lz}$ is the Lempel–Ziv 76 complexity and $l$ is the length (in data samples) of the EEG signal. This entropy rate measures how many bits of innovation are introduced by each data sample and how hard to predict the next. The spectral slope (or the aperiodic component of the EEG power spectrum) was evaluated using the FOOOF^36^ (fitting oscillations and one over *f*, https://fooof-tools.github.io/fooof/) toolbox, with a Python v.3.10 environment. The spectral slope was estimated at the broadband 0.1–32 Hz.

### Dataset cohorts and participant (night) exclusions

We conducted the group-level analysis (Figs. 2 and 3) on the Multi-Ethnic Study of Atherosclerosis (MESA) dataset (cohort 1)^37^ obtained from the National Sleep Research Resources^38^ (http://sleepdata.org). The original aim of the study was to conduct a longitudinal investigation of the sleep-related factors that might lead to cardiovascular diseases in people aged from middle-aged to elderly cohorts. The study contains several examinations spanning several years, and the sleep data were collected during Exam 5 (2010–2013), 10 years after the initial examination. In the first examination, patients with cardiovascular disease were excluded from sampling. During Exam 5, all original participants were invited to the sleep examination, excluding those reporting regular use of oral devices, nocturnal oxygen or nightly positive airway pressure devices. In total, 2,261 finally participated in the sleep examination, with 2,055 participants having overnight home-based polysomnography (PSG) recordings available. All data were collected as part of research protocols that were approved by the local institutional review board at each institution; written, informed consent was obtained from each person before participation. The participants were community-based samples (from six US communities: Baltimore City and Baltimore County, MD; Chicago, IL; Forsyth County, NC; Los Angeles County, CA; Northern Manhattan and the Bronx, NY; and St. Paul, MN). Overnight home-based PSG recordings were recorded using a 15-channel monitor (Compumedics Somte System, Compumedics Ltd). The PSG data are available in European Data Format files along with their annotation files. The sleep stages were scored in 30-s epochs using the AASM criteria. The PSG contained three bipolar-linked EEG recordings: Fz-Cz, Cz-Oz and C4-M1 (Supplementary Fig. 2a). Using the channel signal quality (from one to five, with five indicating the best quality) index provided in the dataset, we excluded the channels with poor channel qualities (smaller than four) within each participant; participants with all channels removed were excluded thoroughly. The beginning of the overnight recording was typically noisy in the first cohort (due to home-based recordings). Therefore, for each EEG channel, the root-mean-square (RMS) values were computed for every 30-s sleep scoring epochs across the whole night, and any 30-s epochs with RMS values greater than two times the median of all epochs were marked as artefactual epochs. This process was repeated twice. Epochs marked artefactual in any channel will also be marked equally in other channels. We cut out initial artefactual data by setting the first artifact-free 30-s sleep scoring epoch as the new beginning of the overnight recordings. For each participant, we extracted their sleep onset period (from the new beginning of recording to sleep onset defined previously) and excluded those with either very short (less than 3 min) or very long (more than 90 min) latency. After participant exclusions, we had *n* = 1,011 participants left for all analyses, with average sleep onset latencies of 48.83 ± 25.01 min (Supplementary Fig. 2b). The remaining participants’ age distribution is shown in Supplementary Fig. 2c,d, grouped by gender (blue for males and orange for females). Specifically, there were 477 men (age 69.81 ± 9.21 years) and 534 women (69.28 ± 8.96 years) and their ages were not different (*P* = 0.45, Kruskal–Wallis test). The sleep latency statistic using the AASM criterion (the beginning of recording to any sleep stage) was 44.15 ± 25.64 mean ± s.d. min (median 44.5 min).

We further analyzed a multinight laboratory-based sleep EEG study in a cohort of healthy young participants for validation and prediction development (cohort 2; Fig. 4). The original aim of the study was to explore the effects of sleep deprivation and circadian rhythms on cognitive performances and the development of biomarkers for individual differences in the regulation of sleep and waking performance, and both the performance^82^ and sleep data^83^ have been published. The research protocol was approved by the Institutional Review Board of the Air Force Research Laboratory and received a favorable opinion from the University of Surrey Ethics Committee. The participants were recruited through flyers, emails, and newspaper and radio advertisements. Inclusion criteria encompassed people in good general health who were free from prescription medications (with the exception of oral contraceptives for female participants). Eligible participants were required to be nonusers of tobacco with moderate caffeine intake patterns (maximum of five caffeinated drinks daily, approximately 500 mg caffeine equivalent) and limited alcohol consumption (not exceeding 14 weekly units). Additional exclusion criteria included self-reported sleep pathology such as sleep apnea or insomnia, current or recent shift work employment and transmeridian travel involving more than one time zone within the 2-month period before laboratory participation. The cohort comprised 36 participants distributed across three *PER3* genotype groups: 12 people with *PER3*4/4, 10 with *PER3*4/5 and 14 with *PER3*5/5 variants. Specifically, the protocol contained two 12-day laboratory sessions, which were separated by at least 10 days. Each session included an adaptation night, a baseline night (with 8 h in bed) and seven nights with either control (that is, extended sleep time to 10 h per 24-h cycle) or sleep restriction (that is, shortened sleep time to 6 h) conditions. After that, there was an additional total sleep deprivation where the participants were asked to stay awake for 39 h (control) or 41 h (sleep restriction) before the last night of sleep in the laboratory (recovery night). More information can be found in the original publications^82,83^. Here we only used the eight nights in the control condition (that is, baseline and the seven sleep extension nights) to focus on natural nocturnal sleep. In total, *n* = 36 participants were tested. The age distribution of the participants can be found in Supplementary Fig. 5a. As in the MESA dataset, we excluded nights with too short latencies (smaller than 3 min), resulting in a total of 267 nights (7.42 ± 0.97 nights per participant). The distribution of sleep onset latency is shown in Supplementary Fig. 5b (42.30 ± 29.25 min). As expected, in the control (sleep extension) condition, the sleep latencies increased in the course of the experiment (Supplementary Fig. 5c) due to the extended time in bed and associated sleep satiation. This will not be further explored in this paper, as we have also shown that the dynamics were independent of the sleep latencies (Extended Data Figs. 1 and 3). PSG recordings were conducted on each night (Siesta 802 devices Compumedics), with an eight-channel EEG montage (F3-M2, F4-M1, C3-M2, C4-M1, P3-M2, P4-M1, O1-M2 and O2-M1) and a sampling rate of 256 Hz. The PSG data were stored in European Data Format files, accompanied by their respective annotation files. The sleep staging was conducted according to the R&K criteria^45^. However, this should not affect our sleep onset definition to any notable extent as the AASM criterion is revised from R&K, with most revisions related to the scoring of slow-wave sleep (stage N3 in AASM, and stage S3 or S4 in R&K)^72^. We mapped the R&K stages S0, S1 and S2 to stages W, N1 and N2 respectively in AASM; R&K stages S3 and S4 to stage N3, as the standard practice. Sleep onset was defined as the first 1 min of stage 2 as before. The sleep latency statistic using the AASM criterion (the beginning of recording to any sleep stage) was 34.27 ± 28.64 min (median 25.5 min).

### EEG preprocessing and transformation into the feature space

Raw EEG data were read out from the PSG for each participant using external toolbox (https://www.edfplus.info/downloads/index.html). We observed substantial cardiac interferences in the EEG signals. Therefore, we first implemented a cardiac interference removal algorithm by applying the method described in ref. ^84^. Briefly, we first extracted the R-peaks in the electrocardiogram (ECG) signal using the Pan-Tompkins method^85^. Then, for each EEG channel, we extracted a 2-s EEG signal aligned to each R-peak detected and averaged all 2-s EEG segments across the night to get a characteristic cardiac signature. We then subtracted the cardiac signature from the EEG for every 2-s segment. More details and the performance evaluation of this method can be found in ref. ^84^.

We then extracted the falling asleep period from bedtime (the beginning of artifact-free recording, see ‘Dataset cohorts and participant (night) exclusions’) to 10 min after sleep onset. The EEG timeseries (every channel) were then segmented into 6-s epochs, with 3-s (50%) overlaps. We computed the feature base of *M* features (cohort 1: 50 features; cohort 2: 47 features) in each epoch. Therefore, for each participant (night), we got an $N\times M\times T$ feature timeseries matrix, where *N* is the number of channels (three in MESA, eight for cohort 2) and *T* is the total number of epochs (timestamps). We then averaged the features across the channels to get representative global brain dynamics or in some cases analyzed each region independently as in Extended Data Fig. 2, and *z*-score normalized the features globally; that is, for each feature, we calculated the mean and s.d. across all epochs of all participants, to which we normalized the features. In this way, we obtained a $M\times T$ feature timeseries matrix representing their dynamics of falling asleep transition in an *M*-dimensional feature space.

To evaluate the feature space dynamics, we normalized the features per participant to the median of the first 10 min after sleep onset (sleep onset state centroid). That is, we took their sleep onset state centroid as the origin of reference for the feature space analysis. The feature state space variables were evaluated accordingly as in the main text. Their vector norms were evaluated as the Euclidean distance in the *M*-dimensional space (MATLAB function pdist). Normalizing the features to the sleep onset state centroid (median of the first 10 min after sleep onset) results in a minimum sleep distance larger than zero. We corrected it by subtracting the minimum sleep distance. In control analysis, where the feature space was reduced to a single feature or a subset of features, all methods remained the same, but only the chosen features were used.

### Group-level analysis of the feature space variables

To ensure data quality for the group-level analysis (Fig. 2), we first applied an artifact rejection to the 6-s epochs. Briefly, for each EEG channel, the RMS values were evaluated for all epochs and the epochs with RMS values above 2.5 the median of all were excluded as artifacts (where their features were marked technically as NaNs). To ensure temporal consistency, if any epoch was marked as an artifact in any channel, the epochs at the same timestamp in the other channels would be excluded as well.

Different participants had different lengths of the falling asleep period, with incomplete artifactual data (NaNs) inside their feature timeseries. We thus took a bootstrapping approach to keep consistent samples at each timestamp to obtain the group-averaged feature timeseries. First, we aligned all participants’ timeseries to their marked sleep onset. The sample number decreases with time going further before the sleep onset point (longer sleep onset latency). We cut off at a point to ensure a minimum of *n* = 200 participant data would be available (around 73.1 min before sleep onset). Then, for each timestamp, we sampled 200 participants’ data randomly to evaluate the group mean feature values. This was done for each feature separately. We repeated the bootstrapping and found that the dynamics were stable with 200 samples. For convenience, we set sleep onset as time zero, and we showed the dynamics starting from 60 min before sleep onset.

The bootstrapping was conducted to derive the group-level dynamics of different feature space variables across all included participants of the MESA dataset (Fig. 2a–c). Specifically, the dynamics were further smoothed using a moving-median window of 20 timestamps (1 min). For the replication in cohort 2 (Extended Data Fig. 7), we conducted bootstrapping across all the selected nights of all participants. Due to the smaller sample size, we choose a minimum of *n* = 150 data samples per timestamp for the group-level dynamics analysis. For these dynamic traces, we did post hoc two-sample *t*-test comparisons (200 samples per timestamp) to the mean of the first 10 min to detect significant changes compared to the baseline. We modified it to be left-tailed or right-tailed based on the observation of whether the dynamic would increase or decrease, and we corrected the significance threshold by dividing the total number of timestamps tested (Bonferroni correction).

### The bifurcation function (model) and its fitting on the sleep distance ${\boldsymbol{s}}({\boldsymbol{t}})$ dynamics

#### The bifurcation function and its fitting

We chose a function (model) that is commonly used to describe a fold (saddle-node) bifurcation due to an overexploitation phenomenon in ecology^40,41^. The overexploitation phenomenon refers to the state (that is, amount) of a natural resource (for example, forest trees) that has an intrinsic growth drive (for example, tree reproduction from pollination) with a limiting growth factor (for example, available land and water) that is exposed to an external reduction drive (for example, tree harvesting). The function (model) describes the rate of change in the amount $x$ of the resource over time, that is, $\frac{{\rm{d}}x}{{\rm{d}}t}$

$$\frac{{\rm{d}}x}{{\rm{d}}t}=rx\left(1-\frac{x}{K}\right)-c\frac{{x}^{2}}{({x}^{2}+{h}^{2})},$$

where $r$ is a parameter representing the resource growth rate (here, tree reproduction), $K$ is a parameter representing the limiting growth factor (here, available land and water), $c$ is a parameter representing the reduction rate (here, tree harvesting) (that is, the control parameter) and $h$ is a parameter that scales down the reduction rate (that is, the control parameter). The equilibrium in the resource amount occurs when $\frac{{\rm{d}}x}{{\rm{d}}t}=0$ (no change over time). In the absence of an external reduction drive ($c=0$), the resource stabilizes at a quantity of $K$; when and more harvesters appear (that is, the control parameter $c$ increases), and the resource biomass would decrease gradually; when there are too many harvesters, the species can no longer sustain reproduction and it will die out catastrophically (bifurcation), with the resource biomass (system state $x$) dropping drastically to the postbifurcation equilibrium state (for example, close to extinction).

In our context of the falling asleep transition, $x$ is the brain (system) state, which we quantified as the sleep distance, and the function (model) describes the rate of change in the distance $s$ from sleep onset over time, that is, $\frac{{\rm{d}}s}{{\rm{d}}t}$. The parameter $r$ represents the rate of distance change away from sleep onset, driven conceptually by an attraction force opposing movement toward sleep onset. The parameter $K$ represents the maximal sleep distance at bedtime. The control parameter $c$ represents the rate of distance change toward sleep onset, driven conceptually by the sleep attraction force (sleep propensity) toward sleep onset. The parameter $h$ then presents as a scaling factor, where smaller values represent a better efficacy of the sleep propensity pushing this transition. Still, the exact interpretation cannot be confirmed without further investigation, potentially with deep-brain activity recordings. We also acknowledge that there could be other dynamical system functions that might describe the process, but the dynamical patterns are widely generalizable across different bifurcation models^86^.

We fitted the function to the empirical sleep distance $s(t)$. Given the set of parameters $r$, $K$, $h$ and m, the two differential equations ($\frac{{\rm{d}}x}{{\rm{d}}t}$ and $\frac{{\rm{d}}c}{{\rm{d}}t}$) were solved using a nonstiff ordinary differential equation solver (MATLAB function ode45), with initial $x$ as the mean of the first 20 values of $s(t)$ timeseries and initial $c=1$. We then optimized the fit of the function $x(t)$ timeseries to the sleep distance $s(t)$ timeseries. We chose two metrics to optimize the function fit: the *R*^2^ value and the RMSE. *R*^2^ was defined as: ${R}^{2}=1-\mathrm{RSS}/\mathrm{TSS}$, where $\mathrm{RSS}$ is the total sum of squares of fit residuals, and $\mathrm{TSS}$ is the total sum of squared target values. The higher the *R*^2^ value, the more variance the model is captured and thus better the function fit. The RMSE is the squared sum of the errors of the fit (between $x(t)$ and $s(t)$) and the lower the value the better the fit. Thus, we set our optimization target to be (${R}^{2}-\mathrm{RMSE})$, where we aim to find the maximum target value for the best fit. We started the optimization by picking a set of initial parameters ($r$, $K$, $h$ and *m*) so that the function can show bifurcation within the same time range as the $s(t)$ timeseries (to ensure that the model started within a critical zone).

The fitting optimization was then executed in a simplified grid search to reduce computational costs. Briefly, we first tuned the parameters $K$ to match the actual system, that is, the mean of the first 100 values of $s(t)$ as an estimation of the system’s carrying capacity (that is, the distance between wakefulness and sleep state in feature space). To ensure that the initial function parameters would still have a critical transition within this time range, the parameters $r$ and $m$ would be up (or down) scaled accordingly (if $K$ decreases or increases). We then applied a grid search to the parameters in a sequential order (from $K$, to $r$, $m$ and finally $h$) to save the computational cost. We started with parameter $K$, where we increased $K$ by 0.25 for ten iterations (with $r$ and $m$ adjusted using the same up/down scaling), and we kept the $K$ value, which would lead to the highest *R*^2^ value. We then initiated a grid search on $r$, $m$ and finally $h$ sequentially. The grid search added or subtracted a small step size (0.02 for $r$, 0.005 for $m$ and 0.001 for $h$) to the initial value to maximize the target value (that is, ${R}^{2}-\mathrm{RMSE}$) over 250 iterations. The fitting accuracy was evaluated as the *R*^2^ values (which is a typical goodness of fit criterion).

The fitted optimal $K$ value was used as an estimation of the sleep distances at bedtime for the evaluation of its correlation with tipping point time. With the optimal parameters set, we then derived the bifurcation diagram by solving the differential equation $\frac{{\rm{d}}x}{{\rm{d}}t}=0$ (the $r$, $K$ and $h$ are constants, whereas $c$ changes over time with rate $m$). The roots of the equation over time form the bifurcation diagram (Fig. 2e). The critical zone has three solutions typically, with two stable attractors (system states) and one unstable. The tipping point can be identified at the end of this three-solution period, that is, the system attractors would collapse to one. In practice, there would be cases where no critical zone can be found. In this case, we defined it as a fitting failure.

#### Fitting on the group-level s(*t*) dynamics

The group-averaged sleep distance $s(t)$ timeseries were derived from bootstrapping (see above), and fitting used the stated optimization method. The bifurcation diagram with the optimal fit parameters and the extracted tipping point were then derived as described above.

#### Fitting on the individual-level *s*(*t*) dynamics

The individual-level sleep distance $s(t)$ dynamics were evaluated as described above. To ensure data continuity, no artifact exclusion was done to the individual-level traces. The fitting was highly dependent on whether we could get a good sleep onset centroid representation (Extended Data Fig. 1a); therefore, we further excluded participants (or nights) with a mean sleep score of the first 10 min after sleep onset smaller than 1.5 (an objective noncomputational factor). We also showed that the bedtime–sleep distance also impacts the fitting accuracy (Extended Data Fig. 1b); however, this is a computational factor and, therefore, we did not make exclusions of participants based on this factor. This resulted in *n* = 626 in the MESA and *n* = 267 nights in cohort 2.

For each sleep distance $s(t)$ timeseries, we focused on the last 30 min (or less if the latency was smaller than 30) before sleep onset as there were barely any changes in the dynamics before this time, and we applied a causal running window median filtering (that is, only using values in the past) with a window size of 20 to smooth the timeseries. We then fitted the bifurcation function to the individual-level $s(t)$ traces, with relevant bifurcation diagrams and tipping points evaluated as described above. In cases where the fit did not have a critical zone (see above), we defined it as a bifurcation fit failure and excluded these participants and nights. In the MESA dataset, only 23 out of 626 (3.6%) failed to fit, and 11 out of 267 nights failed (4.1%) in cohort 2.

For the individual-level analysis within individual regions in the first cohort (MESA, frontal and occipital regions specifically), we further excluded participants with bad channel qualities in either region (quality index smaller than four, see previous), resulting in *n* = 549. In the frontal region, 42 (7.6%) had a bifurcation fit failure, with 23 (4.2%) participants in the occipital region. After excluding these participants, in total *n* = 496 participants were used for statistical analysis in the main text.

### Evaluation of early warning signals of critical slowing down on sleep distance *s*(*t*) dynamics

Critical slowing down is a specific phenomenon that happens when a dynamical system is driven to bifurcation at a moderate speed (that is, change of underpinning control parameter)^33,87^. During the transition toward the tipping point, the system will become disturbed more easily (a weaker attractor), which is reflected in the dynamics of the system as of a larger variance and autocorrelation. These properties are therefore called the early warning signals (EWS) and can help predict the tipping point (critical transition) before it happens. Critical slowing down has been studied extensively in ecological systems^88^, financial markets^89^ and so on. In the neuroscience field, it has been shown that critical slowing down can be observed before transitions into a seizure state^86^.

#### Evaluations of EWS on sleep distance *s*(*t*)

There is a rich repertoire of EWS methods to detect upcoming tipping points in a data-driven way; here we focused on the two most common: autocorrelation and variance. The implementation of EWS computation followed the methods in ref. ^52^. On the sleep distance $s(t)$ timeseries, autocorrelation and variance were evaluated in a running window with a window size of 100 samples (5 min). In each running window, we first detrended the timeseries linearly (using MATLAB function detrend), and the residuals after detrending were taken to evaluate the autocorrelation (MATLAB function autocorr) and variance (MATLAB function std). This detrending is important to avoid spurious increases in autocorrelation and variance due to changes in the mean values of the timeseries^52^.

The same set of participants was included (*n* = 626, see previous section for participant exclusions) for this analysis. We evaluated the EWS on the sleep distance $s(t)$ timeseries of each person (the 30 min before sleep onset plus 10 min after). This would return two EWS timeseries (autocorrelation and variance) that were 5 min shorter due to the initial running window. We then used the same bootstrapping strategy to obtain the group-averaged autocorrelation and variance timeseries traces (Fig. 2f), with each timestamp having 200 samples. Similarly, significant changes in the EWS values were tested against the mean of the first 3-min period, using the right-tailed two-sample *t*-test with Bonferroni correction. To determine whether the variance and autocorrelation increased constantly over time (before bifurcation, that is, sleep onset here) statistically, we further fitted a linear mixed effect model with the EWS values as the response variable and time as the predictor, and the participant as random effects. The analysis of variance was used to analyze the model statistics, where the coefficient and *P* value of the ‘Time’ variable determined whether the linear increase was significant over time. The EWSs were also compared to the sleep stages, and the changes were tested against the mean value of the first 3 min to identify the timestamp at which they started to increase significantly.

To ensure that the observed increases in EWSs were related to sleep onset transition rather than artifacts, we selected another set of participants with sleep latency above 55 min (*n* = 425) and took the first 30 min of their $s(t)$ timeseries, that is, the sleep distance timeseries were at least 25 min away before sleep onset. We applied the same analysis as for Fig. 2f and no increases in either of the EWS indexes with time were found (Supplementary Fig. 4b).

### FPCA for key feature dynamics

FPCA is a dimensionality reduction tool designed for functional data observed continuously in space and time^43^ (Fig. 3a–f). FPCA is an extension of the Karhunen–Loève transform into discrete timeseries data. FPCA was implemented using the existing Python toolbox ‘scikit-fda’ (https://fda.readthedocs.io/en/latest/index.html#, v.0.7), in a Python v.3.8 environment.

#### Implementation of FPCA on feature timeseries

The feature timeseries for each participant was evaluated (described previously), *z*-score normalized globally and normalized to the median of the 10-min sleep onset (shifted origin). The same bootstrapping was applied here to derive the group-averaged feature timeseries (for all 50 features, *n* = 200 per timestamp). We then extracted only the pre-asleep period for FPCA analysis. FPCA with Ridge regularization was then applied to the 50 group-level feature timeseries. The first two FPCs were chosen given that they explained over 99% of the variance. The FPCs were presented by addition (positive, +) or subtraction (negative, −) to the mean of all feature timeseries as positive and negative directions.

To select the representative features for each FPC, we used the FPC scores returned from the method fit_transform, and then normalized the scores to [−1,1]. Briefly, for positive (or negative) scores, we divided the scores by the maximum (or minimum) score values. The features with a normalized score larger than 0.9 (or smaller than −0.9) were selected as representative features on the positive (or negative) side.

#### Find representative features for the control parameter

The control parameter timeseries derived from the optimal model fit on the group level (Fig. 2a) was compared to the 50 feature timeseries. The best representative feature should have a linear increasing pattern with the closest slope (rate) to that of the control parameter. Therefore, we used two metrics: the absolute cosine similarity (which evaluates the timeseries shape similarity) and the DTW distance (the distance in rate). Cosine similarity was evaluated as $\frac{{x}_{{ft}}\left(t\right)\bullet c\left(t\right)}{{||}{x}_{{ft}}(t){||}\times {||c}\left(t\right){||}}$, where ${x}_{{ft}}\left(t\right)$ is the feature timeseries, and $c\left(t\right)$ is the control parameter timeseries. The higher the absolute cosine similarity, and the lower the DTW distance, the better the features could represent the control parameter. Therefore, we designed a metric as the absolute cosine similarity divided by the DTW distance, and the feature with the highest metric value was chosen as the representative feature of the control parameter. DTW was calculated using MATLAB function dtw.

### Real-time prediction of the sleep distance *s*(*t*) timeseries based on the training night

The key knowledge for estimating the $s(t)$ was the sleep onset centroid, to which the feature space was referenced as the origin. Therefore, we first evaluated the sleep onset state in the feature space across all nights of each participant and evaluated their consistency using the Silhouette score. Silhouette score measures how similar the data within each cluster are compared to other clusters, and ranges from −1 to 1 (the higher the value, the better the clustering). Briefly, the sleep-onset centroids (median of the 10-min postsleep-onset period, 199 data samples) were evaluated for every night of all participants, and the Silhouette score was evaluated using the participant ID as the cluster indices (to cluster the centroids). The score was then compared to those of 1,000 surrogates (generated by random shuffling of the participant index per night). The *P* value was calculated as *C*/1,000, where *C* is the total number of surrogates having a higher Silhouette score value than the original (better clustered in surrogates). A *P* value smaller than 0.05 indicates significantly good clustering with original cluster indices (that is, the sleep onset centroids were consistent within this participant).

To predict the sleep distance traces, we first randomly selected a night as training (repeated five times per participant). We evaluated the sleep onset centroid (the median of the post-10-min period) in the feature space as the trained sleep onset state. As described previously, we also recorded the computational gap that normalizes the minimum $s(t)$ to zero. For the testing nights, for each incoming EEG epoch (6 s, with a 3-s gap between them), we evaluated the 47 features, *z*-score normalized them to the mean and s.d. values from the training night, and then computed the Euclidean distance to the trained sleep onset centroid (with the gap correction). To reduce noise, we applied a causal moving-median filter of window size 20 data samples. Typically, these can be done within 0.5 s (less than the 3-s gap). We did that for all other testing nights of the participant. To evaluate the performance of prediction (accuracy), we compared the predicted and the actual $s(t)$ (derived post hoc) using the cosine similarity metric (same as before). The methods are the same for selecting several nights as training. The results shown were collated from all five random repetitions.

### Real-time prediction of the transition tipping point

For the training night, we evaluated the sleep distance $s(t)$ referenced to the sleep-onset centroid in the feature space from this night only, and we fitted the function toward the timeseries and derived the tipping point. We further recorded the $s(t)$ value at which the tipping point was set as the critical value. On the testing nights, in a quasi-real-time way, we monitored the predicted $s(t)$ and marked the locations where this critical value was crossed (in the downward direction) as a potential predicted tipping point.

To evaluate the performance of such a prediction, we observed post hoc the entire predicted sleep distance $s(t)$ traces of the testing nights (until 10 min after the marked sleep onset), and we kept only those downward crossings that remained below the critical value for longer than 1 min as valid predictions (crossings). We further defined the predicted tipping point as the last crossing without further upward crossing back, where earlier crossings were defined as erroneous early predictions. The predicted tipping points were then compared to the actual tipping point given by the function fit (evaluated post hoc on the actual $s(t)$).

The tipping points were quantified against the hypnogram sleep onset post hoc. If the tipping points were identified after the sleep onset, we defined them as invalid tipping points and excluded them from analysis. Across all the nights, 11 out of 267 (4.1%) nights failed fitting, which were excluded from the prediction analysis. Across all the predictions (with five random repetitions), 13% of the cases (141 nights out of 1085) had the predicted tipping point after sleep onset, and we excluded them before further quantifications against the actual model.

### Reporting summary

Further information on research design is available in the Nature Portfolio Reporting Summary linked to this article.

## Online content

Any methods, additional references, Nature Portfolio reporting summaries, source data, extended data, supplementary information, acknowledgements, peer review information; details of author contributions and competing interests; and statements of data and code availability are available at 10.1038/s41593-025-02091-1.

## Supplementary information

Supplementary Information Supplementary note (text), Figs. 1–7 and Tables 1–6.

Reporting Summary

## Figures and Tables

**Extended Data Table 1:**
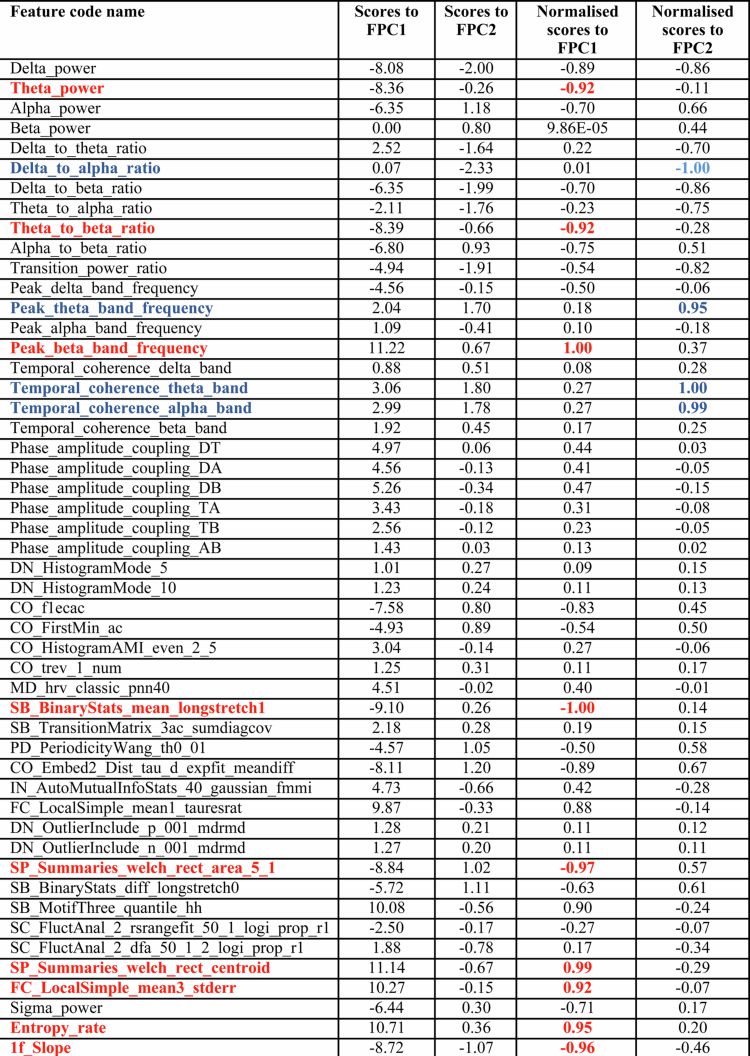
FPC scores of the features (related to Fig. 3)

The representative features for FPC1 are marked in bold and red, and for FPC2 marked in blue.

**Extended Data Table 2:**
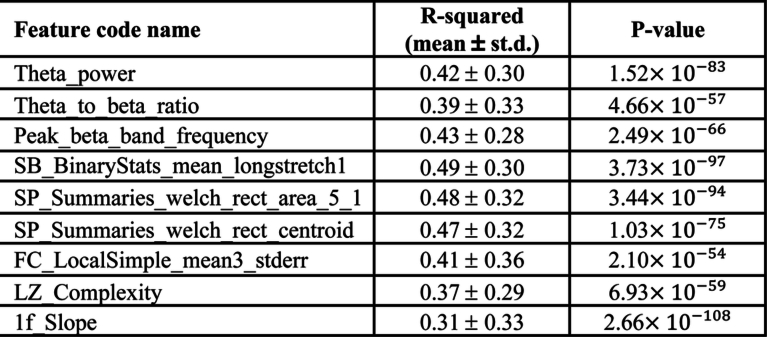
The average R-squared across the group for bifurcation function fitting on the individual participant’s FPC1 representative features time series (absolute feature values)

The p-value was evaluated using the right-sided one-sample t-test of the R-squared differences between fittings on individual features and fittings on the sleep distance.

**Extended Data Table 3:**
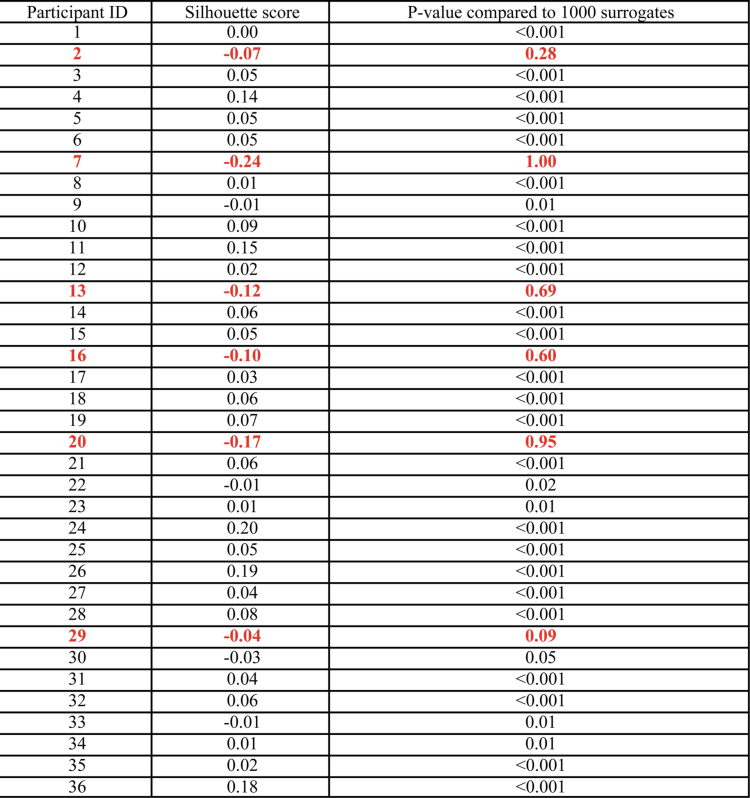
The Silhouette scores and *P*-values for clustering the sleep onset centroids of different nights within each participant (related to Fig. 4)

*P*-value smaller than 0.05 indicates the sleep-onset centroids across different nights were clustered together. Participants with inconsistent centroids are marked in bold and red.

## Data Availability

Cohort 1 is based on the Multi-Ethnic Study of Atherosclerosis (MESA) open-source dataset that can be obtained from National Sleep Research Resources (NSRR); https://sleepdata.org/datasets/mesa. Cohort 2 can be shared under a material transfer agreement by contacting D.-J.D., d.j.dijk@surrey.ac.uk. At the time of the study, participants were not asked to provide consent to share or deposit anonymised raw data; however, they were informed that the results would be published only in anonymized form. Therefore, public repository deposition of the data has not been possible. The timeframe for responding to data access requests will normally be within 2 weeks. The results data to reproduce all the figures is available on GitHub (https://github.com/Jlkkkcc/Paper-FallingAsleepBifurcation).

## Code availability

Codes generated in this paper are available on GitHub (https://github.com/Jlkkkcc/Paper-FallingAsleepBifurcation). The repository includes: all the process scripts from raw data processing to final results; the final data and codes for recreating all main and extended data figures in the paper; and the introductory examples for the key algorithms and methods implemented. Documentations are also provided in the repository.
