# Supplementary material for: Falling asleep follows a predictable bifurcation dynamic
Source: Nat Neurosci. 2025 Oct 28;28(12):2515–25. doi: 10.1038/s41593-025-02091-1 (PMC12672367; doi:10.1038/s41593-025-02091-1)
Supplement: Supplementary file 1 — Supplementary note (text), Figs. 1–7 and Tables 1–6. [file 41593_2025_2091_MOESM1_ESM.pdf]

nature neuroscience

Supplementary information

https://doi.org/10.1038/s41593-025-02091-1

Falling asleep follows a predictable bifurcation dynamic

In the format provided by the authors and unedited

Supplementary Materials

Supplementary Notes

Fold (saddle-node) bifurcation

A bifurcation is a 'qualitative' shift of the system's state or behaviour in response to a smooth and gradual change in the (control) parameter of a dynamical system. The type of qualitative change (bifurcation) depends on how the system's equilibria (a.k.a. steady states where the system can remain, in our case, wakefulness or sleep states in the brain) are created or destroyed or change in their stability. The fold bifurcation is a specific type of bifurcation, where the gradual change in the control parameter causes the original stable equilibrium to disappear, or more formally, collide with another non-observable (i.e., exists theoretically) unstable equilibrium and annihilate. This will lead the system to have an abrupt discontinuous transition to another state, sometimes also described as a catastrophic regime shift. The point at which this bifurcation occurs is also known phenomenologically as the tipping point (or the point of no return). A simple, illustrative example would be bending a stick (see illustration in Fig.S1): we start with no force and gradually increase our force (control parameter) to bend it; the stick will show minor deformation until at some point, when the force is strong enough (at the tipping point), the stick will snap, which is a catastrophic change into a new system state with the original state disappeared. This is an extreme example where the transition is not reversible; in many natural systems, however, the bifurcations are reversible, and they can follow a different return path, thus leading to hysteresis[1].

Earlier experimental evidence of a falling asleep bifurcation

To our knowledge, this is the first experimental evidence that the brain's falling asleep phenomenon displays a bifurcation dynamic. Our findings address the long-lasting gap in our understanding of the falling asleep dynamic as a continuous process supporting the bifurcation concept hypothesised using neural circuit modelling. We note a recent case report (two human participants) that reported the feasibility of early warning signals (such as EEG autocorrelation and variance) shortly (~25s) before different sleep stage transitions, although not between wake and sleep[2]. We also note a recent study that showed critical slowing down in an autoregressive integrated moving average model of heart rate variability as participants stopped responding to instructions (button press) while falling asleep (no brain activity was measured)[3].

Earlier demonstration of real-time prediction of progression into sleep

To our knowledge, this is the first demonstration of real-time prediction of the brain's progression into sleep. We note the report by Prerau et al.[4], who derived a probability of losing behavioural response while falling asleep, quantified by the accuracy of pressing a stress ball during breath inhales. The authors derived a Bayesian model summing a state process based on a muscle's electromyography (EMG) amplitude during ball pressing and state processes based on the brain's alpha and delta-theta band powers. The authors showed a large inter-subject heterogeneity in the behavioural loss, but improved tracking accuracy overall compared to normal sleep stages. Yet, using an active task such as ball-pressing to quantify the falling asleep process can bias the natural phenomenon[5]. In addition, the changes in behavioural response do not represent the complex changes in brain activity during falling asleep. Other reports, such as those by Strauss et al.[6] and Jagannathan et al.[7] linked a set of EEG features to subgroups of Hori micro-states[8] (manually scored) and vigilance (assessed using auditory evoked potential or choice response task). The computational framework described in this paper is distinct from these earlier works. Instead of linking changes in brain activity to changes in behavioural response[4] or Hori micro-states[6,7] during the falling asleep process, we describe the changes in brain activity themselves. We also note the recent report by Haasan et al. that focused on changes in brain activity[9]. The authors clustered three states, i.e., wakefulness, drowsiness, and sleep during falling asleep (wakefulness and N1 stages), using a sigmoid-shaped wakefulness probability function derived from a weighted sum of normalised changes in EEG band powers (delta, alpha, and beta). Our description of the changes in brain activity goes beyond discrete states, providing a continuous measure of the sleep-relevant changes with a defined wake–sleep cutoff. In contrast to earlier

approaches, our descriptive model is entirely data-driven without an assumption on the governing features or a need for hyperparameter fiddling. Finally, we note prediction attempts using other physiological measures, such as heart rate variability[3], respiratory[10], and body temperature[11] (alas, without benchmarking to brain EEG changes or sleep staging). However, these physiological metrics do not capture the high temporal dynamics of the brain falling asleep[12]. Hence, they may support brain-based measures but not replace them. This would be especially the case in patients with sleep-onset disorders such as paradoxical insomnia[13].

Supplementary Figures

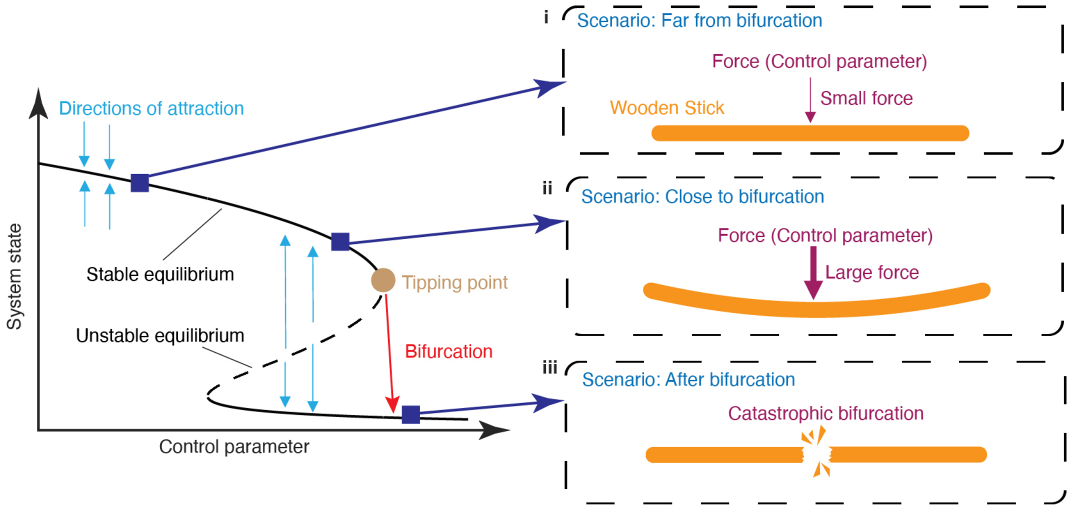

Fig.S1. Illustration of the bifurcation process via the bending stick example.

A representative fold (saddle-node) bifurcation diagram(left) and the analogy of a bifurcation process during the bending of a stick (right). In the bifurcation diagram, the solid line shows the stable equilibria, while dashed lines show unstable ones. The light blue arrows show the directions of attraction (i.e., where the system states would go if they were not at equilibrium). The tipping point (orange dot) is where the stable and unstable equilibria collide and annihilate, leading to a bifurcation into a completely different state (the bottom equilibrium).

The bending stick bifurcation process shows the changes in the system state (stick integrity) as a function of the control parameter (bending force). When the force is small, the stick shows minor or no deformation ((i), far from bifurcation). With the gradual increase of the force, the stick shows larger deformation (ii). When the force surpasses the tipping point value, bifurcation happens, and the stick would break (iii), with the original state (a complete stick) disappearing.

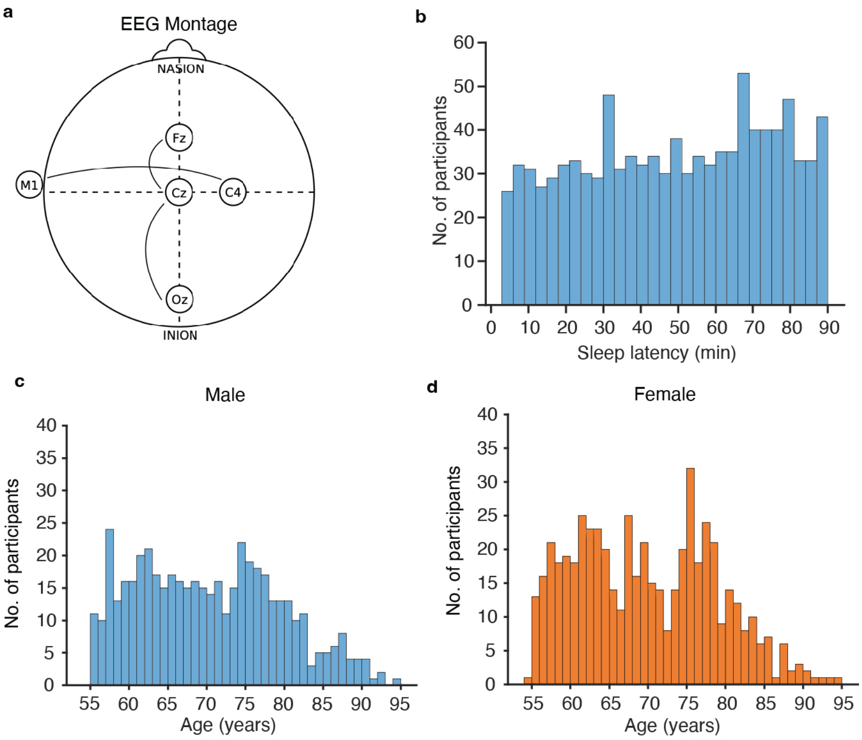

Fig.S2. Cohort 1 information (related to Fig.2-3).

Cohort 1 is from the Multi-Ethnic Study of Atherosclerosis (MESA) dataset[14,15].

a, Recording electrode montage, including Fz-Cz, Cz-Oz and C4-M1 derivations of the 10-20 system.

b, Distribution of participants' sleep onset latency (n = 1011).

c, Distribution of male participants' age (n = 477).

d, Distribution of female participants' age (n = 534).

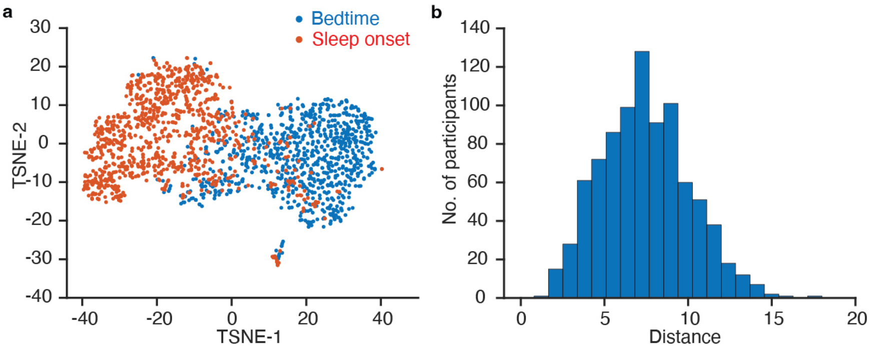

Fig.S3. The distinctiveness of bedtime and sleep onset states in the feature space (related to Fig.2).

a, Distribution of the participant's bedtime state (orange) and sleep onset state (blue) centroids, visualised in a 2-dimensional (2D) using t-distributed stochastic neighbour embedding (t-SNE).

b, Histogram of participant's Euclidean distances between bedtime and sleep-onset states in the feature space.

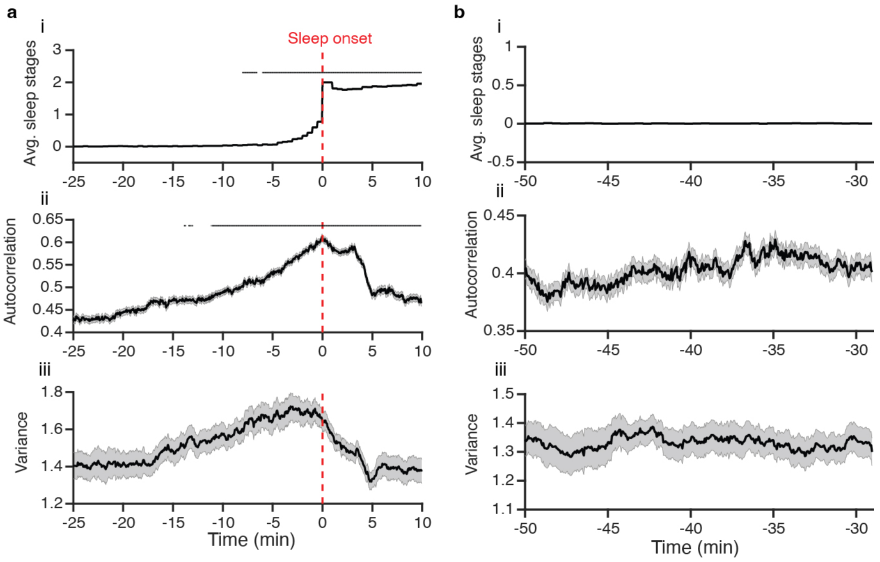

Fig.S4. Critical slowing down and early warning signals (related to Fig.2).

a, Group-averaged (i) sleep stage, (ii) autocorrelation, and (iii) variance of the participants' sleep distance $s(t)$ time series computed from 30 minutes before to 10 minutes after sleep onset. Showing mean value (black line) and standard error of the mean, s.e.m., (shaded areas). Horizontal lines at the top of the panel indicate a significant increase compared to the first 3 minutes computed using a right-tailed two-sample t-test with a p-value threshold Bonferroni corrected for the number of timestamps. Vertical red dashed line indicates sleep onset time. Linear mixed effect model: For autocorrelation, the Linear mixed effect model coefficient (with time as predictor and participant index as random effect) was 0.006, $F_{(1,2.52\times10^5)} = 268.84$, p = 2.19× 10^{-60}. For Variance, coefficient 0.008, $F_{(1,3.52\times10^5)} = 31.85$, p = 1.69× 10^{-8} (n = 626).

b, Same as **a** but for a period without falling asleep transition (see Methods for details). Linear mixed effect model: For autocorrelation, the Linear mixed effect model coefficient was 0.0004, $F_{(1,2.21\times10^5)} = 0.87$, p = 0.35. For Variance, coefficient -0.0009, $F_{(1,2.07\times10^5)} = 0.52$, p = 0.47 (n = 425).

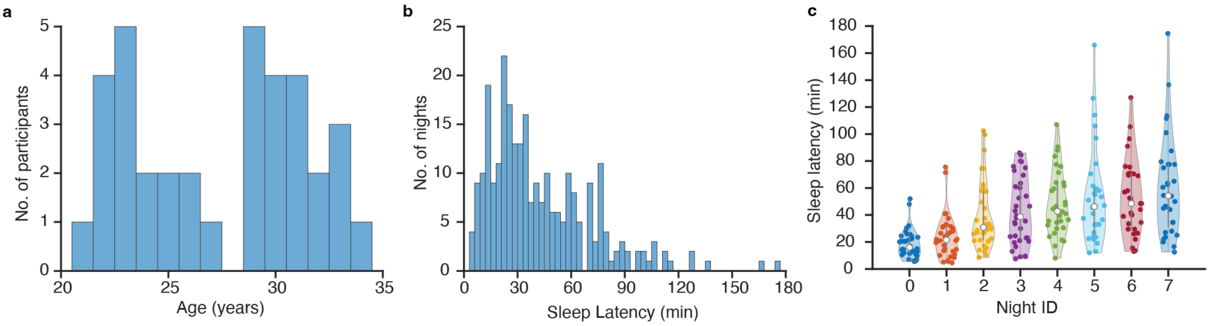

Fig.S5. Cohort 2 information (related to Fig.4).

a, Distribution of participants' age (n = 36).

b, Distribution of all participants' sleep latency (n = 267).

c, Sleep latency distribution grouped by night number (i.e., the day of the lab visit). The violins show the kernel density (the shape), the median values (white non-filled circle) and upper and lower 25% quantiles (grey bar). Refer to Table S6 for their statistics.

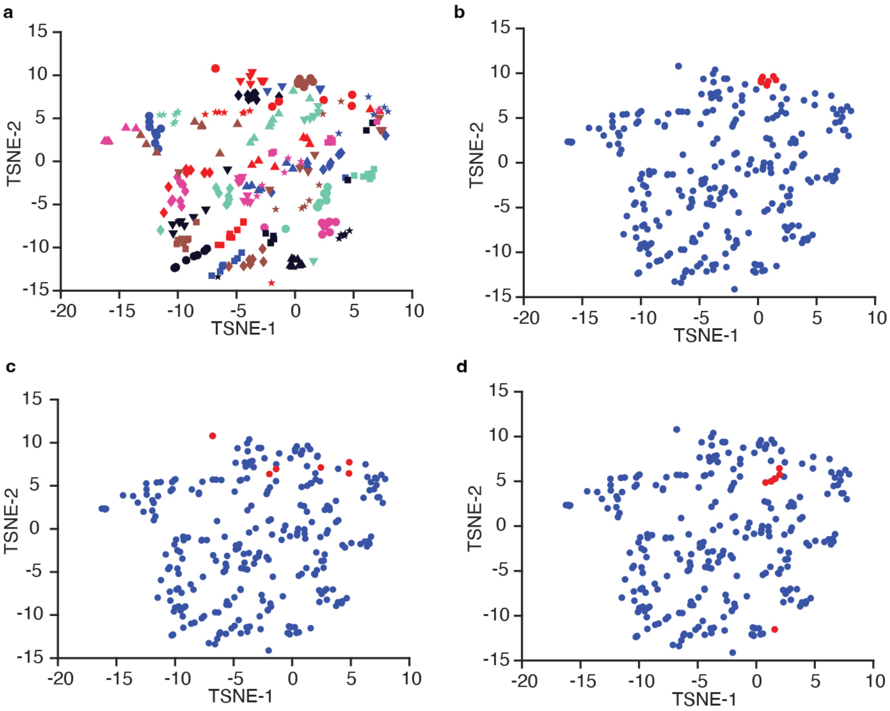

Fig.S6. Two-dimensional distribution of individual sleep onset centroids computed using t-SNE (related to Fig.4).

a, 2-D view of the participants' feature space sleep onset centroids. Each colour-marker pair denotes nights from the same participants (n = 267 nights for n = 36 participants in total). The x and y axes are t-SNE embedding dimensions.

b, Same as **a,** but with a representative participant with consistent sleep onset centroid across nights in red (tested via the Silhouette score test) and the remaining participants in blue.

c, Same as **b,** but for a participant without a consistent sleep onset centroid.

d, Same as **b,** but for a participant with most of the nights consistent, except one.

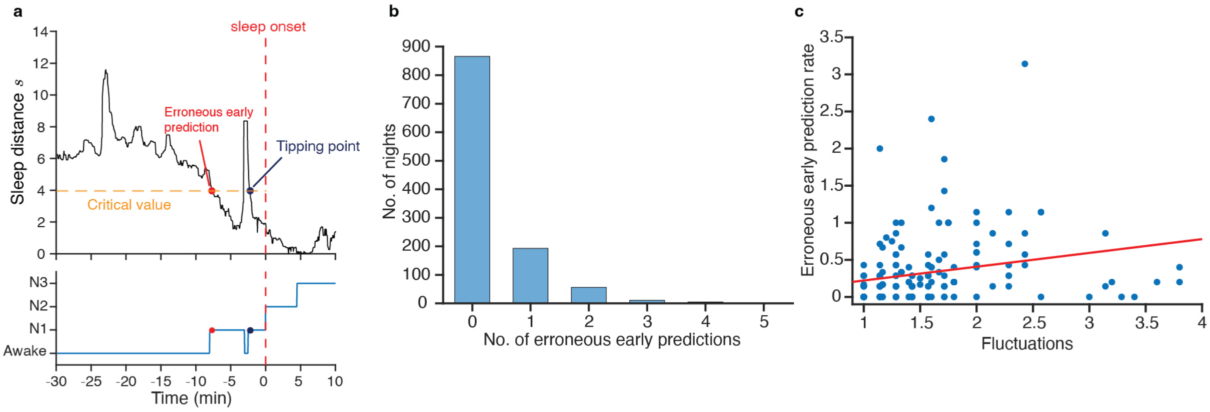

Fig.S7. Cases of erroneous early tipping point prediction (related to Fig.4).

a, An exemplary case of erroneous early tipping point prediction. The blue circular marker indicates the tipping point, and the red circular marker indicates an earlier crossing of the sleep distance critical value. The orange horizontal line shows the critical threshold determined by the training model. The red vertical dashed line shows the sleep onset. The bottom panel shows the corresponding hypnogram of that night, with the red and blue tipping point markers copied.

b, Histogram of the number of erroneous early tipping point predictions across all the testing nights (with random training night picked and repeated five times).

c, Correlation between the probability of erroneous early tipping point prediction (averaged per training case, in total n = 180) and the hypnogram fluctuations (i.e., number of sleep stage transitions). The red line shows the linear regression line, r = 0.24, p = 0.001, two-sided Pearson correlation test (n = 179).

Supplementary Tables

Table S1. List of EEG time series features used for analysis

List 1: Conventional EEG analysis features (25 in total)

Feature code name	*Descriptions*
**Power-band features**	
Delta_power	The mean delta band (0.5 – 4 Hz) power normalized to the entire spectrum power and log transformed
Theta_power	The mean theta band (4 – 8 Hz) power normalized to the entire spectrum power and log transformed
Alpha_power	The mean alpha band (8 – 12 Hz) power normalized to the entire spectrum power and log transformed
Beta_power	The mean beta band (12 – 30 Hz) power normalized to the entire spectrum power and log transformed
Delta_to_theta_ratio	The log-transformed band power ratio between delta and theta band
Delta_to_alpha_ratio	The log-transformed band power ratio between delta and alpha band
Delta_to_beta_ratio	The log-transformed band power ratio between delta and beta band
Theta_to_alpha_ratio	The log-transformed band power ratio between theta and alpha band
Theta_to_beta_ratio	The log-transformed band power ratio between theta and beta band
Alpha_to_beta_ratio	The log-transformed band power ratio between delta and alpha band
Transition_power_ratio	The log-transformed transition power ratio defined as the low frequency band power (delta plus theta) divided by high frequency band power (alpha plus beta)
Peak_delta_band_frequency	The peak frequency in the delta band (frequency bin with highest power)
Peak_theta_band_frequency	The peak frequency in the theta band (frequency bin with highest power)
Peak_alpha_band_frequency	The peak frequency in the alpha band (frequency bin with highest power)
Peak_beta_band_frequency	The peak frequency in the beta band (frequency bin with highest power)
**Temporal coherence features**	
Temporal_coherence_delta_band	The temporal coherence in delta band
Temporal_coherence_theta_band	The temporal coherence in theta band
Temporal_coherence_alpha_band	The temporal coherence in alpha band
Temporal_coherence_beta_band	The temporal coherence in beta band
**Phase-amplitude coupling features**	
Phase_amplitude_coupling_DT	The phase-amplitude coupling strength between delta band phase and theta band amplitude
Phase_amplitude_coupling_DA	The phase-amplitude coupling strength between delta band phase and alpha band amplitude
Phase_amplitude_coupling_DB	The phase-amplitude coupling strength between delta band phase and beta band amplitude
Phase_amplitude_coupling_TA	The phase-amplitude coupling strength between theta band phase and alpha band amplitude
Phase_amplitude_coupling_TB	The phase-amplitude coupling strength between theta band phase and beta band amplitude

Phase_amplitude_coupling_AB	The phase-amplitude coupling strength between alpha band phase and beta band amplitude

List 2: Catch-22: canonical highly comparative time-series features (Reused from[16] under Creative Commons Attribution 4.0 International License).

Feature code name	Descriptions
**Distribution**	
DN_HistogramMode_5	Mode of z-scored distribution (5-bin histogram)
DN_HistogramMode_10	Mode of z-scored distribution (10-bin histogram)
**Simple temporal statistics**	
SB_BinaryStats_mean_longstretch1	Longest period of consecutive values above the mean
DN_OutlierInclude_p_001_mdrmd	Time intervals between successive extreme events above the mean
DN_OutlierInclude_n_001_mdrmd	Time intervals between successive extreme events below the mean
**Linear autocorrelation**	
CO_f1ecac	First $1/e$ crossing of autocorrelation function
CO_FirstMin_ac	First minimum of autocorrelation function
SP_Summaries_welch_rect_area_5_1	Total power in lowest 1/5 of frequencies in the Fourier power spectrum
SP_Summaries_welch_rect_centroid	Centroid of the Fourier power spectrum
FC_LocalSimple_mean3_stderr	Mean error from a rolling 3-sample mean forecasting
**Nonlinear autocorrelation**	
CO_trev_1_num	Time-reversibility statistic, $\langle (x_{t+1} - x_t)^3 \rangle_t$
CO_HistogramAMI_even_2_5	Automutual information, m=2, τ=5
IN_AutoMutualInfoStats_40_gaussian_fmmi	First minimum of the automutual information function
**Successive differences**	
MD_hrv_classic_pnn40	Proportion of successive differences exceeding 0.04σ
SB_BinaryStats_diff_longstretch0	Longest period of successive incremental decreases
SB_MotifThree_quantile_hh	Shannon entropy of two successive letters in equiprobable 3-letter symbolization
FC_LocalSimple_mean1_tauresrat	Change in correlation length after iterative differencing
CO_Embed2_Dist_tau_d_expfit_meandiff	Exponential fit to successive distances in 2-d embedding space
**Fluctuation Analysis**	
SC_FluctAnal_2_dfa_50_1_2_logi_prop_r1	Proportion of slower timescale fluctuations that scale with DFA (50% sampling)
SC_FluctAnal_2_rsrangefit_50_1_logi_prop_r1	Proportion of slower timescale fluctuations that scale with linearly rescaled range fits
**Others**	
SB_TransitionMatrix_3ac_sumdiagcov	Trace of covariance of transition matrix between symbols in 3-letter alphabet
PD_PeriodicityWang_th0_01	Periodicity measure of [17]

List 3: Additional specific EEG characterisations related to sleep (3 features)

Feature code name	Descriptions
**Power-band features**	
Sigma_power	The mean sigma band (12-16 Hz) power normalized to the entire spectrum power and log transformed; This band is the one within which spindles happen.
LZ_Complexity	A normalised version of Lempel-ziv complexity (the entropy rate estimation of the EEG signal) is implemented, which estimates how many bits of innovation are introduced by each data sample, and how hard to predict the next[18]; This has

	also been shown to be better than directly using the Lempel-ziv complexity measurement[19].30/08/2025 22:44:00
1f_Slope	The 1/f slope (aperiodic component) of the EEG power spectrum, estimated using the FOOOF toolbox[20] at a broadband (0.1-32 Hz). The output feature values were reflected, where a larger value indicates a sharper slope (i.e., more power at low-frequency parts).

Table S2. Interpretation of the representative FPC features mentioned in the main text.

Feature code name	Name used in text	Explanations
Theta_power	Theta band power	As described in the method;
Delta_to_alpha_ratio	Delta-to-alpha ratio	As described in the method;
Theta_to_beta_ratio	Theta-to-beta ratio	As described in the method;
Peak_theta_band_frequency	Peak theta frequency	As described in the method;
Peak_beta_band_frequency	Peak beta frequency	As described in the method;
Temporal_coherence_theta_band	Theta temporal coherence	As described in the method;
Temporal_coherence_alpha_band	Alpha temporal coherence	As described in the method;
SB_BinaryStats_mean_longstretch1	Dwelling time	A catch22 feature evaluated as the longest period of time that the EEG data would be above its mean. The slower the EEG is fluctuating around its mean (e.g., slower frequency), the larger the feature value.
SP_Summaries_welch_rect_area_5_1	Total spectrum power	This feature captures the first fifth total spectrum power; Given that our EEG sampling rate is 256Hz, the first fifth of the spectrum ranges from 0 to around 25Hz; In the typical scenario of sleep EEG the spectrum power is mostly concentrated below 30Hz, so we interpret it as approximately the 'total spectrum power'.
SP_Summaries_welch_rect_centroid	Spectrum centroid	This feature measures the centroid of the EEG power spectrum, estimating where the spectrum energy is concentrated. Lower feature values imply that the energy is concentrated in lower frequency bands.
FC_LocalSimple_mean3_stderr	Prediction Error	This feature measures the error for predicting future 3 samples within the EEG signal; The larger the value, the lower predictable the EEG will be; for instance, a slow changing EEG will be very predictable (thus low feature values).
LZ_Complexity	Lempel-Ziv Complexity	As described in the method;
1f_Slope	Spectral slope	As described in the method;

Table S3. The cosine similarity and DTW distances between the control parameter and the features (related to Fig.3).

The best representative feature is marked in red.

Feature code name	DTW distance	Cosine similarity	Similarity / DTW
Delta_power	66.23	0.94	0.014
Theta_power	66.22	0.88	0.013
Alpha_power	65.19	0.79	0.012
Beta_power	42.72	0.52	0.012
Delta_to_theta_ratio	31.32	0.13	0.004
Delta_to_alpha_ratio	49.07	0.76	0.016
Delta_to_beta_ratio	66.03	0.96	0.014
Theta_to_alpha_ratio	63.26	0.97	0.015
Theta_to_beta_ratio	66.33	0.90	0.014
Alpha_to_beta_ratio	65.53	0.81	0.012
Transition_power_ratio	65.66	0.96	0.015
Peak_delta_band_frequency	66.07	0.88	0.013
Peak_theta_band_frequency	11.31	0.82	0.073
Peak_alpha_band_frequency	39.85	0.43	0.011

Peak_beta_band_frequency	13.28	0.88	0.066
Temporal_coherence_delta_band	31.88	0.13	0.004
**Temporal_coherence_theta_band**	**6.69**	**0.94**	**0.140**
Temporal_coherence_alpha_band	7.10	0.93	0.132
Temporal_coherence_beta_band	14.53	0.74	0.051
Phase_amplitude_coupling_DT	13.50	0.83	0.061
Phase_amplitude_coupling_DA	14.73	0.80	0.054
Phase_amplitude_coupling_DB	17.05	0.78	0.046
Phase_amplitude_coupling_TA	15.77	0.75	0.048
Phase_amplitude_coupling_TB	19.01	0.68	0.036
Phase_amplitude_coupling_AB	28.53	0.25	0.009
DN_HistogramMode_5	33.11	0.16	0.005
DN_HistogramMode_10	30.51	0.14	0.005
CO_f1ecac	65.69	0.82	0.013
CO_FirstMin_ac	65.24	0.80	0.012
CO_HistogramAMI_even_2_5	16.71	0.72	0.043
CO_trev_1_num	27.92	0.23	0.008
MD_hrv_classic_pnn40	14.93	0.82	0.055
SB_BinaryStats_mean_longstretch1	66.08	0.86	0.013
SB_TransitionMatrix_3ac_sumdiagcov	14.69	0.81	0.055
PD_PeriodicityWang_th0_01	64.91	0.78	0.012
CO_Embed2_Dist_tau_d_expfit_meandiff	65.40	0.80	0.012
IN_AutoMutualInfoStats_40_gaussian_fmmi	21.55	0.70	0.032
FC_LocalSimple_mean1_tauresrat	16.95	0.83	0.049
DN_OutlierInclude_p_001_mdrmd	29.87	0.20	0.007
DN_OutlierInclude_n_001_mdrmd	30.52	0.17	0.006
SP_Summaries_welch_rect_area_5_1	65.63	0.82	0.012
SB_BinaryStats_diff_longstretch0	65.14	0.79	0.012
SB_MotifThree_quantile_hh	17.47	0.81	0.047
SC_FluctAnal_2_rsrangefit_50_1_logi_prop_r1	65.91	0.89	0.014
SC_FluctAnal_2_dfa_50_1_2_logi_prop_r1	33.42	0.12	0.004
SP_Summaries_welch_rect_centroid	17.78	0.81	0.046
FC_LocalSimple_mean3_stderr	15.54	0.84	0.054
Sigma_power	66.04	0.85	0.013
LZ_Complexity	13.97	0.86	0.062
1f_Slope	66.42	0.91	0.014

Table S4. The mean Silhouette scores and number of participants with consistent sleep onset centroids with only one feature instead of the feature space (related to Fig.4)

**Feature code name**	**Silhouette scores (mean ± std across all participants)**	**No. participants with consistent centroids ($p<0.05$) out of 36 participants**
Theta_power	-0.42 ± 0.32	2 (5.5%)
Delta_to_alpha_ratio	-0.38 ± 0.28	0 (0%)
Peak_beta_band_frequency	-0.42 ± 0.32	1 (2.7%)
SB_BinaryStats_mean_longstretch1	-0.48 ± 0.34	3 (8.3%)
SP_Summaries_welch_rect_area_5_1	-0.47 ± 0.26	0 (0%)
SP_Summaries_welch_rect_centroid	-0.46 ± 0.33	2 (5.5%)
FC_LocalSimple_mean3_stderr	-0.45 ± 0.30	2 (5.5%)

Table S5. Statistics of cosine similarity for different numbers of training nights used (related to Fig.4bii)

No. of training nights	Cosine similarity (mean ± st.d.)
1	0.95 ± 0.06
2	0.97 ± 0.03
3	0.97 ± 0.03
4	0.97 ± 0.03
5	0.97 ± 0.04
6	0.98 ± 0.02
7	0.98 ± 0.03

Table S6. Sleep latency statistics per night visit for the second cohort (related to Fig.S5)

Night ID	Mean latency (min)	St.d.
1	18.81	10.78
2	23.93	16.26
3	39.49	24.69
4	43.31	24.54
5	48.53	23.39
6	53.25	35.62
7	53.11	28.00
8	60.83	37.09

References

1. Luppi, A. I., Spindler, L. R. B., Menon, D. K. & Stamatakis, E. A. The Inert Brain: Explaining Neural Inertia as Post-anaesthetic Sleep Inertia. *Frontiers in Neuroscience* **15**, (2021).
2. de Mooij, S. M. M. *et al.* Dynamics of sleep: Exploring critical transitions and early warning signals. *Computer Methods and Programs in Biomedicine* **193**, 105448 (2020).
3. Demareva, V. *et al.* Forecasting the transition to sleep through HRV analysis: insights from ARIMA analysis and the concept of critical slowing down. *Biological Rhythm Research* **55**, 159–169 (2024).
4. Prerau, M. J. *et al.* Tracking the sleep onset process: an empirical model of behavioral and physiological dynamics. *PLoS computational biology* **10**, (2014).
5. Curcio, G., Casagrande, M. & Bertini, M. Sleepiness: evaluating and quantifying methods. *International Journal of Psychophysiology* **41**, 251–263 (2001).
6. Strauss, M., Sitt, J. D., Naccache, L. & Raimondo, F. Predicting the loss of responsiveness when falling asleep in humans. *NeuroImage* **251**, (2022).
7. Jagannathan, S. R. *et al.* Tracking wakefulness as it fades: Micro-measures of alertness. *NeuroImage* **176**, 138–151 (2018).
8. Hori, T., Hayashi, M. & Morikawa, T. Topographical EEG changes and the hypnagogic experience. in *Sleep onset: Normal and abnormal processes* 237–253 (1994).
9. Hassan, A. R., Kabir, M., Keshavarz, B., Taati, B. & Yadollahi, A. Sigmoid Wake Probability Model for High-Resolution Detection of Drowsiness Using Electroencephalogram. in *2019 41st Annual International Conference of the IEEE Engineering in Medicine and Biology Society (EMBC)* 7080–7083 (2019). doi:10.1109/EMBC.2019.8857801.
10. Guede-Fernández, F., Fernández-Chimeno, M., Ramos-Castro, J. & García-González, M. A. Driver Drowsiness Detection Based on Respiratory Signal Analysis. *IEEE Access* **7**, 81826–81838 (2019).
11. Partonen, T., Haukka, J., Kuula, L. & Pesonen, A.-K. Assessment of time window for sleep onset on the basis of continuous wrist temperature measurement. *Biological Rhythm Research* **53**, 897–907 (2022).
12. Ogilvie, R. D. The process of falling asleep. *Sleep Medicine Reviews* **5**, 247–270 (2001).
13. Rezaie, L., Fobian, A. D., McCall, W. V. & Khazaie, H. Paradoxical insomnia and subjective–objective sleep discrepancy: A review. *Sleep Medicine Reviews* **40**, 196–202 (2018).
14. Chen, X. *et al.* Racial/Ethnic Differences in Sleep Disturbances: The Multi-Ethnic Study of Atherosclerosis (MESA). *Sleep* **38**, 877–888 (2015).
15. Zhang, G. Q. *et al.* The National Sleep Research Resource: Towards a sleep data commons. *Journal of the American Medical Informatics Association* **25**, 1351–1358 (2018).
16. Lubba, C. H. *et al.* catch22: CAnonical Time-series CHaracteristics. *Data Mining and Knowledge Discovery* **33**, 1821–1852 (2019).
17. Wang, X., Wirth, A. & Wang, L. Structure-Based Statistical Features and Multivariate Time Series Clustering. in *Seventh IEEE International Conference on Data Mining (ICDM 2007)* 351–360 (2007). doi:10.1109/ICDM.2007.103.
18. Mediano, P. A. M. *et al.* Effects of External Stimulation on Psychedelic State Neurodynamics. *ACS Chem. Neurosci.* **15**, 462–471 (2024).
19. Mediano, P. A. M. *et al.* Spectrally and temporally resolved estimation of neural signal diversity. 2023.03.30.534922 Preprint at https://doi.org/10.1101/2023.03.30.534922 (2023).
20. Donoghue, T. *et al.* Parameterizing neural power spectra into periodic and aperiodic components. *Nature Neuroscience* **23**, 1655–1665 (2020).
