# Supplementary material for: Falling asleep follows a predictable bifurcation dynamic
Source: Nat Neurosci. 2025 Oct 28;28(12):2515–25. doi: 10.1038/s41593-025-02091-1 (PMC12672367; doi:10.1038/s41593-025-02091-1)
Supplement: Supplementary file 2 — Reporting Summary [file 41593_2025_2091_MOESM2_ESM.pdf]

nature portfolio

Corresponding author(s): Nir Grossman, Junheng Li

Last updated by author(s): Aug 29, 2025

Reporting Summary

Nature Portfolio wishes to improve the reproducibility of the work that we publish. This form provides structure for consistency and transparency in reporting. For further information on Nature Portfolio policies, see our Editorial Policies and the Editorial Policy Checklist.

Statistics

For all statistical analyses, confirm that the following items are present in the figure legend, table legend, main text, or Methods section.

n/a	Confirmed	
☐	☒	The exact sample size (*n*) for each experimental group/condition, given as a discrete number and unit of measurement
☐	☒	A statement on whether measurements were taken from distinct samples or whether the same sample was measured repeatedly
☐	☒	The statistical test(s) used AND whether they are one- or two-sided<br>*Only common tests should be described solely by name; describe more complex techniques in the Methods section.*
☒	☐	A description of all covariates tested
☐	☒	A description of any assumptions or corrections, such as tests of normality and adjustment for multiple comparisons
☐	☒	A full description of the statistical parameters including central tendency (e.g. means) or other basic estimates (e.g. regression coefficient) AND variation (e.g. standard deviation) or associated estimates of uncertainty (e.g. confidence intervals)
☐	☒	For null hypothesis testing, the test statistic (e.g. *F*, *t*, *r*) with confidence intervals, effect sizes, degrees of freedom and *P* value noted<br>*Give P values as exact values whenever suitable.*
☒	☐	For Bayesian analysis, information on the choice of priors and Markov chain Monte Carlo settings
☒	☐	For hierarchical and complex designs, identification of the appropriate level for tests and full reporting of outcomes
☐	☒	Estimates of effect sizes (e.g. Cohen's *d*, Pearson's *r*), indicating how they were calculated

Our web collection on statistics for biologists contains articles on many of the points above.

Software and code

Policy information about availability of computer code

Data collection	N.A.
Data analysis	The pre-processing, feature extraction, model fitting (algorithms), and post-hoc statistical analysis were mostly done in MATLAB R2023a (Ubuntu system); Only the Functional Principal Component Analysis (FPCA) was done using Python3.8, Scipy (Version 1.6.2), Scikit-fda (Version 0.7). External MATLAB software used for EEG feature extraction: The CATCH-22 (https://github.com/DynamicsAndNeuralSystems/catch22), v0.1.0 was used. For the normalised Lempel-Ziv complexity feature (Entropy rate), the EntRate toolbox was used (https://github.com/pmediano/EntRate). For the aperiodic exponent feature, the FOOOF toolbox (python) was used (https://fooof-tools.github.io/fooof/#), but the python function was called in MATLAB, with the supporting python version 3.10.<br>For plotting: ShadedErrorBar (https://uk.mathworks.com/matlabcentral/fileexchange/26311-raacampbell-shadederrorbar); ViolinPlot (https://github.com/bastibe/Violinplot-Matlab); Colors (https://uk.mathworks.com/matlabcentral/fileexchange/29702-generate-maximally-perceptually-distinct-colors?#functions_tab).<br>The raw sleep data (PSG format) reading used external toolbox: https://www.edfplus.info/downloads/index.html.<br>All other methods were developed in-house, and the detailed methodologies can be found in the manuscript.<br>All process codes (from raw sleep data to final results), final data and codes to reproduce the figures, and the introductory examples of the key algorithms in the paper has been published on GitHub (https://github.com/Jlkkkcc/Paper-FallingAsleepBifurcation).

For manuscripts utilizing custom algorithms or software that are central to the research but not yet described in published literature, software must be made available to editors and reviewers. We strongly encourage code deposition in a community repository (e.g. GitHub). See the Nature Portfolio guidelines for submitting code & software for further information.

Data

Policy information about availability of data

All manuscripts must include a data availability statement. This statement should provide the following information, where applicable:

- Accession codes, unique identifiers, or web links for publicly available datasets
- A description of any restrictions on data availability
- For clinical datasets or third party data, please ensure that the statement adheres to our policy

Cohort 1 is based on the Multi-Ethnic Study of Atherosclerosis (MESA) open-source dataset that can be obtained from National Sleep Research Resources (NSRR); https://sleepdata.org/datasets/mesa. Cohort 2 can be shared under a material transfer agreement by contacting D.J.D., d.j.dijk@surrey.ac.uk. At the time of the study, participants were not asked to provide consent to share or deposit anonymised raw data; however, they were informed that the results would be published only in anonymised form. Therefore, public repository deposition of the data has not been possible. The timeframe for responding to data access requests will normally be within 2 weeks. The results data to reproduce all the figures is available on GitHub (https://github.com/Jlkkkcc/Paper-FallingAsleepBifurcation).

Research involving human participants, their data, or biological material

Policy information about studies with human participants or human data. See also policy information about sex, gender (identity/presentation), and sexual orientation and race, ethnicity and racism.

Reporting on sex and gender	Gender information of the MESA dataset was provided along with sleep data via National Sleep Research Resources (NSRR). The gender information for the second cohort was collected during the experiment. The gender information was collected all with participants' consents. Gender distribution was reported, MESA: 524 females, 487 males. For the second cohort: 18 females, 18 males. No gender-grouped analysis was done and reported.
Reporting on race, ethnicity, or other socially relevant groupings	Race and ethnicity data was provided along with sleep data via National Sleep Research Resources (NSRR). Race and ethnicity information was collected in the second cohort during the experiment. All information was collected with participants' consents. No analyses was done, nor did any conclusions was drawn, based on these variables. No race or ethnicity information was reported.
Population characteristics	The original aim of the study was to conduct a longitudinal investigation of the sleep-related factors that might lead to cardiovascular diseases in people aged from middle-aged to elderly cohorts. The study contains multiple examinations spanning several years, and the sleep data was collected during Exam 5 (2010-2013), 10 years after the initial examination. In the first examination, CVD patients were excluded from sampling, and a total of 6,814 men and women who identified themselves as White, Black/African American, Hispanic, or Chinese aged 45–84 were recruited. 38 percent of the recruited participants were White, 28 percent African American, 22 percent Hispanic, and 12 percent Chinese. The first examination took place over two years, from July 2000 - July 2002. During Exam 5, all original participants were invited to the sleep examination, excluding those reporting regular use of oral devices, nocturnal oxygen, or nightly positive airway pressure (PAP) devices. A total of 4,077 participants were approached; 147 (6.5%) were ineligible (95 due to a history of the PAP use (2%); 4 due to use of an oral appliance; and 4 due to oxygen use) and 141 participants lived too far away to participate. 2,261 participated in the sleep exam (59.7%) Of the remaining 3,789, with 2055 participants having overnight home-based polysomnography (PSG) recordings available on NSRR. Please refer to original publication for more details: Xiaoli Chen, Rui Wang, Phyllis Zee, Pamela L. Lutsey, Sogol Javaheri, Carmela Alcántara, Chandra L. Jackson, Michelle A. Williams, Susan Redline, Racial/Ethnic Differences in Sleep Disturbances: The Multi-Ethnic Study of Atherosclerosis (MESA), Sleep, Volume 38, Issue 6, 1 June 2015, Pages 877–888, https://doi.org/10.5665/sleep.4732<br>Among the 2055 participants with overnight PSG data, further exclusion was applied based on data qualities (based on data quality and the purpose of this study about falling asleep dynamics), and n=1011 participants were included in the final analysis: 524 females, age 69.4 ± 9.07 mean ± st.d. years. No genotypic information was provided. The participants were heterogeneous with no specific exclusion for disease-related criteria in Exam 5; only those participants reporting regular use of oral devices, nocturnal oxygen, or nightly positive airway pressure (PAP) devices were excluded . See below recruitment for exclusion details.<br><br>The participants included in the second cohort: n=36 subjects, 18 females; age 27.42 ± 4.02 mean ± st.d. years. A buccal swab was obtained from each participant to determine their PER3 genotype. The 36 participants distributed across three PER3 genotype groups: 12 individuals with PER34/4, 10 with PER34/5, and 14 with PER35/5 variants. All participants were healthy individuals; see below for further inclusion/exclusion criteria during recruitment.
Recruitment	For the MESA cohort, the participants were diverse community-based samples from from six US communities: Baltimore City and Baltimore County, MD; Chicago, IL; Forsyth County, NC; Los Angeles County, CA; Northern Manhattan and the Bronx, NY; and St. Paul, MN. Each of six sites aimed to recruit 1,100 participants, equally divided between men and women. Wake Forest, Johns Hopkins, Minnesota, and Northwestern all started by creating community awareness of the study and enlisting the support and endorsement of community-based organizations and leadership. All sites implemented techniques that have been used successfully in other studies to recruit minority populations. Columbia worked closely with the 1199 National Benefit Fund during recruitment, including using study staff hired through the union for recruitment, retention, and study publicity. UCLA recruited using random-digit dialing. All sites that recruited Hispanics employed staff fluent in Spanish, and sites recruiting Chinese-Americans employed staff fluent in Cantonese and Mandarin. Prior to recruitment, the purpose, rationale, and design of the study were publicized to residents of target areas at each site. Successive efforts were directed at targeted individuals, and included mailings of letters and brochures, followed by personal contacts via telephone or in person. Sites modified these materials to meet unique aspects of the source population and recruitment strategy. Standard press releases were written, and templates were developed for participant letters, brochures, and scripts.<br><br>For the second cohort, participants were recruited through flyers, emails, and newspaper and radio advertisements. Inclusion

	criteria encompassed individuals in good general health who were free from prescription medications (with the exception of oral contraceptives for female participants). Eligible participants were required to be non-tobacco users with moderate caffeine intake patterns (maximum of five caffeinated drinks daily, approximately 500 mg caffeine equivalent) and limited alcohol consumption (not exceeding 14 weekly units). Additional exclusion criteria included self-reported sleep pathology such as sleep apnea or insomnia, current or recent shift work employment, and transmeridian travel involving more than one time zone within the two-month period prior to laboratory participation. For further information, please refer to: Lo, June C., et al. "Effects of partial and acute total sleep deprivation on performance across cognitive domains, individuals and circadian phase." (2012): e45987.
Ethics oversight	Ethical approval for the analysis of sleep data of human participants was granted by the Imperial College Research Ethics Committee (ICREC). For the MESA cohort, all data were collected as part of research protocols that were approved by the local institutional review board at each institution. For the second cohort, the research protocol was approved by the Institutional Review Board of the Air Force Research Laboratory and received a favourable opinion from the University of Surrey Ethics Committee. Written, informed consent was obtained from each individual before participation.

Note that full information on the approval of the study protocol must also be provided in the manuscript.

Field-specific reporting

Please select the one below that is the best fit for your research. If you are not sure, read the appropriate sections before making your selection.

☒ Life sciences ☐ Behavioural & social sciences ☐ Ecological, evolutionary & environmental sciences

For a reference copy of the document with all sections, see nature.com/documents/nr-reporting-summary-flat.pdf

Life sciences study design

All studies must disclose on these points even when the disclosure is negative.

Sample size	Sample size was not determined a priori based on statistical considerations, and our main results were generated from the MESA cohort, with sufficiently large sample size (n>1000), which is considerably larger than most other sleep EEG studies.<br><br>We then replicated and extended the analysis in cohort 2. We chose the cohort 2 data primarily due to its richness, with multiple continuous laboratory-based overnight sleep PSG recordings for the same individual, which provided the possibility for us to run individual sleep-onset prediction analysis.
Data exclusions	For the MESA dataset, participants with poor EEG data quality and sleep-onset latencies outside the range of 3-90 minutes were excluded (1044 in total); For the second cohort, no participants were excluded, we only included in the analysis 8 sleep extension nights, with remaining sleep restricted nights excluded. Further exclusions apply during the analysis, due to failures in fitting the bifurcation function or other reasons to ensure robust results. All further exclusions were stated in the methods.
Replication	The main analysis was done on the MESA cohort. The computational results were then replicated on the Cohort 2. The analysis were taken further in cohort 2 for individual prediction analysis, given the richness of the data which contained multiple nights per participant. The individual predictions used one night from each individual as the training night, to predict the remaining nights' sleep onset of the same individual. This prediction was replicated five times, with each replication taking one random night (out of all available nights of the same individual) as the training night.
Randomization	Randomization was not relevant to this study, as there was no experimental or therapeutic group/condition.
Blinding	N.A. Our computational analysis reported did not involve any comparisons between treatment / experimental groups.

Reporting for specific materials, systems and methods

We require information from authors about some types of materials, experimental systems and methods used in many studies. Here, indicate whether each material, system or method listed is relevant to your study. If you are not sure if a list item applies to your research, read the appropriate section before selecting a response.

Materials & experimental systems

n/a	Involved in the study	
☒	☐	Antibodies
☒	☐	Eukaryotic cell lines
☒	☐	Palaeontology and archaeology
☒	☐	Animals and other organisms
☒	☐	Clinical data
☒	☐	Dual use research of concern
☒	☐	Plants

Methods

n/a	Involved in the study	
☒	☐	ChIP-seq
☒	☐	Flow cytometry
☒	☐	MRI-based neuroimaging

Plants

Seed stocks	N.A.
Novel plant genotypes	N.A.
Authentication	N.A.
